# Immune-regulating strategy against rheumatoid arthritis by inducing tolerogenic dendritic cells with modified zinc peroxide nanoparticles

**DOI:** 10.1186/s12951-022-01536-0

**Published:** 2022-07-14

**Authors:** Han Qiao, Jingtian Mei, Kai Yuan, Kai Zhang, Feng Zhou, Tingting Tang, Jie Zhao

**Affiliations:** 1 Department of Orthopaedic Surgery, Shanghai Ninth People’s Hospital, Shanghai Jiao Tong University School of Medicine, 639 Zhizaoju Road, Shanghai, 200011 People’s Republic of China; 2 Shanghai Key Laboratory of Orthopaedic Implants, Shanghai Ninth People’s Hospital, Shanghai Jiao Tong University School of Medicine, Shanghai, People’s Republic of China; 3 Department of Orthopaedic Surgery, The First Affiliated Hospital of Soochow University, Suzhou, Jiangsu Province People’s Republic of China

**Keywords:** Nanoimmunotherapy, Tolerogenic dendritic cell, Zinc and oxygen homeostasis, Rheumatoid arthritis, Zinc peroxide

## Abstract

In hypoxic dendritic cells (DCs), a low level of Zn^2+^ can induce the activation of immunogenic DCs (igDCs), thereby triggering an active T-cell response to propel the immune progression of rheumatoid arthritis (RA). This finding indicates the crucial roles of zinc and oxygen homeostasis in DCs during the pathogenesis of RA. However, very few studies have focused on the modulation of zinc and oxygen homeostasis in DCs during RA treatment. Proposed herein is a DC-targeting immune-regulating strategy to induce igDCs into tolerogenic DCs (tDCs) and inhibit subsequent T-cell activation, referred to as ZnO_2_/Catalase@liposome-Mannose nanoparticles (ZnCM NPs). ZnCM NPs displayed targeted intracellular delivery of Zn^2+^ and O_2_ towards igDCs in a pH-responsive manner. After inactivating OTUB1 deubiquitination, the ZnCM NPs promoted CCL5 degradation via NF-κB signalling, thereby inducing the igDC-tDC transition to further inhibit CD4^+^ T-cell homeostasis. In collagen-induced arthritis (CIA) mice, this nanoimmunoplatform showed significant accumulation in the spleen, where immature DCs (imDCs) differentiated into igDCs. Splenic tDCs were induced to alleviate ankle swelling, improve walking posture and safely inhibit ankle/spleen inflammation. Our work pioneers the combination of DC-targeting nanoplatforms with RA treatments and highlights the significance of zinc and oxygen homeostasis for the immunoregulation of RA by inducing tDCs with modified ZnO_2_ NPs, which provides novel insight into ion homeostasis regulation for the treatment of immune diseases with a larger variety of distinct metal or nonmetal ions.

**Graphical Abstract:**

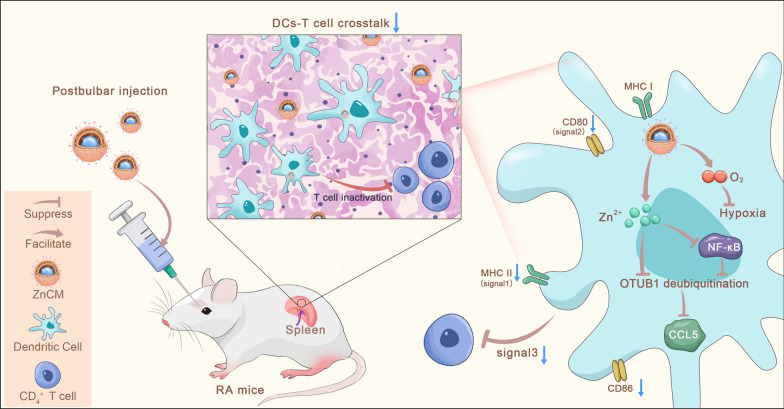

**Supplementary Information:**

The online version contains supplementary material available at 10.1186/s12951-022-01536-0.

## Highlights

- The DC-targeting immune-regulating nanostrategy was firstly employed to treat RA.
- The complex immune regulating effects was realized through a portable, convenient and green nanomaterial.
- Highlighting the significance of zinc and oxygen homeostasis for the immunoregulation of RA by inducing tDCs with modified ZnO_2_ NPs.
- Expanding the notion of ion homeostasis regulation with a larger variety of distinct metal or nonmetal ions.

**Supplementary Information:**

The online version contains supplementary material available at 10.1186/s12951-022-01536-0.

## Introduction

The significant specialized roles of dendritic cells (DCs) in the pathogenesis of rheumatoid arthritis (RA) have long been acknowledged [1, 2]. RA is a common inflammatory autoimmune systematic disease that mainly contributes to progressive ankylosis, deformity or luxation of the affected joints [3–5]. Previously, it was shown that DCs were the major antigen-presenting cells (APCs) that initiated the priming and differentiation of effector CD4^+^ T cells to activate the adaptive immune response in RA [6]. Immature DCs (imDCs) are a class of bone marrow-derived cells that largely migrate towards the spleen for further differentiation into immunogenic DCs (igDCs). Splenic igDCs exhibit systemic immune effects, including inflammation of the ankle synovium in RA [7]. Specifically, nonself extracellular RA antigens are initially differentiated by splenic imDCs based on the associated surface immunostimulatory signals, followed by the subsequent activation of imDCs into igDCs for antigen presentation, which facilitates the systemic activation and maturation of downstream CD4^+^ T cells. Herein, igDCs secreted a variety of inflammatory cytokines against T cells to bolster the particular importance of DC-T-cell homeostasis during RA progression [8]. It was demonstrated that clinical remission with immune-suppressive candidates could decrease the matured level of igDCs and alleviate RA advancement significantly [9, 10], implying the potential application of DC-targeted therapeutics for RA treatment.

The concept of ion homeostasis articulates the significance of intracellular ions on cellular fate by introducing ions (including Ca^2+^) into cells, which could affect biological behaviours [11]. Herein, intracellular Zn^2+^ homeostasis orchestrates the activation and maturation of DCs that are vital for the oriented T-cell response [12]. It was shown that lipopolysaccharide (LPS) stimulation of DCs resulted in decreased levels of Zn^2+^ importer Zrt/Irt-like protein 6 (ZIP6) and increased expression of Zn^2+^ exporters via TLR-4, contributing to the accelerated activation of igDCs with downregulation of the intracellular Zn^2+^ concentration [13, 14]. Conversely, imDCs supplemented with an excess of Zn^2+^ were induced to develop a tolerogenic DC (tDC) phenotype, leading to the reduced maturation of DCs and their antigen presentation abilities towards T cells [12, 15]. Hence, it is of great significance to efficiently regulate DC intracellular Zn^2+^ homeostasis, thereby modulating essential immune functions with a decreased risk of excessive inflammation [16]. More importantly, the low oxygen state, which is characterized as the main feature of RA progression [17], was found to likewise influence DC migration, antigen presentation and activation [18]. The costimulatory molecules CD80 and CD86 on the DC surface were upregulated after exposure to hypoxia [19], indicating the potent role of hypoxia in igDC activation to promote RA. Therefore, the intracellular homeostasis of Zn^2+^ and O_2_ in DCs has been suggested to control the functions of the essential immune response, thus indicating the direction of development for DCs immunotherapy.

Innovative solutions to target and regulate DC behaviors are expected to tremendously facilitate clinical immunotherapeutic strategies for RA. Nanomedicine combined with immunotherapy aimed at inhibiting immune cell activation or recruitment via an appropriate drug delivery platform might represent a potent intervention approach to fight diseases [20, 21]. For instance, nanoproteoliposomes were deployed as nanocarriers for the antigen GM3 ganglioside in renal carcinoma-bearing mice, demonstrating that these nanoproteoliposomes could significantly transform the splenic DC phenotype towards APCs, followed by inhibition of the immune-suppressive characteristics of splenic DCs to promote subsequent cytotoxic T-cell activation [22, 23]. Moreover, polymeric nanoparticles loaded with CpG or paclitaxel were found to effectively target DCs within lymph nodes, which drastically reduced DC immune activity [24]. Nonetheless, to date, such DC-targeting nanostrategies have mainly focused on the development of anticancer therapeutics, while a limited number of reports have covered the combination of nanomedicine with immunotherapy for RA treatment. Hence, since RA is a common autoimmune disorder in the clinic, this prompted us to exploit the possible benefits of targeting DCs for nanoimmunotherapy against RA.

Herein, acid-degradable zinc peroxide nanoparticles (ZnO_2_ NPs) were first synthesized to shuttle extra Zn^2+^ towards DCs. DC-targeting ligand mannose (man)-anchored liposomes were further applied to coencapsulate ZnO_2_ NPs with catalase (Cat) to form the final biofunctional ZnO_2_/Cat@lipo-mannose (ZnCM) NPs [25–27]. This approach not only alleviates DC hypoxia but also avoids undesired Zn^2+^ release. After their specific uptake by DCs, modified ZnO_2_ NPs decomposed in a pH-responsive manner in the lysosomal acidic microenvironment to release Zn^2+^ and H_2_O_2_ [28]. H_2_O_2_ can be further catalysed by Cat to produce oxygen (O_2_), which alleviates the intracellular hypoxic state and assists Zn^2+^ in promoting the DC transition into tDCs [29–31], thereby attenuating the immune destruction of RA.

The DC-targeted immune-regulating nanostrategy was prepared in a green and convenient way and applied to RA treatment for the first time. We expect such an immune nanoplatform of both Zn^2+^ and O_2_ homeostasis regulation could exert tolerogenic effects on DCs in RA, thus expanding the notion of ion homeostasis to include immune diseases in addition to cancers with a larger variety of distinct metal or nonmetal ions.

## Results and discussion

### Synthesis and characterization of the nanoparticles

**Fig. 1:**
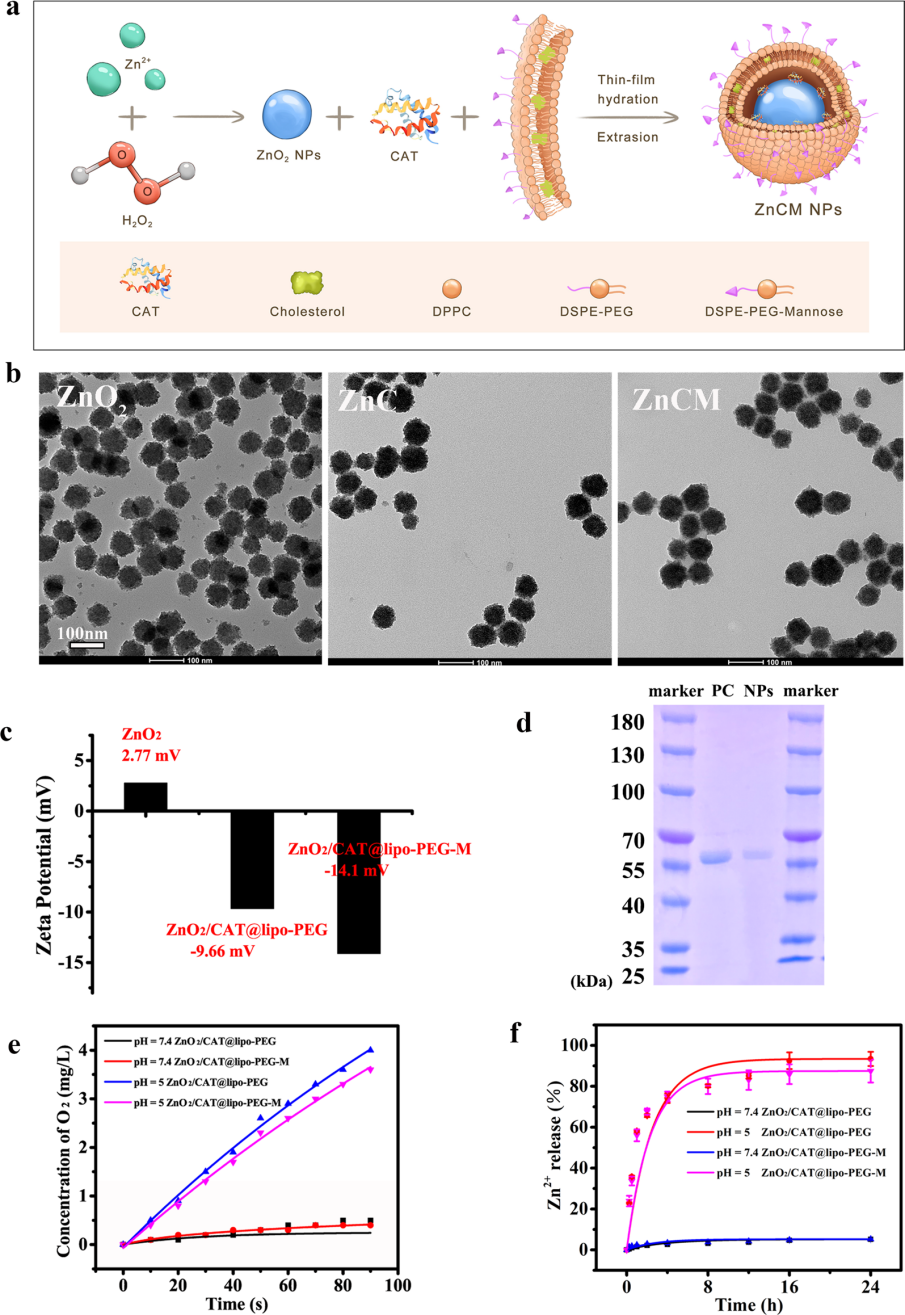
Characterizations of modified ZnO_2_ NPs. **a** Schematic illustration of modified ZnO_2_ NPs synthesis. **b** TEM images of ZnO_2_, ZnC and ZnCM NPs. **c** Zeta potentials of different NPs. **d** SDS-PAGE gel analysis of Cat (lane 1: positive control, PC, of free Cat; lane 2: ZnCM NPs). **e** Concentration changes of dissolved O_2_ from ZnC and ZnCM NPs under different pH values. **f** Percentage changes of released Zn^2+^ ion from ZnC and ZnCM NPs under different pH values

The processes to synthesize the modified Zn^2+^ NPs were illustrated in Fig. 1a. ZnO_2_ NPs were first prepared by a wet chemistry synthesis method. XRD patterns (Additional file 1: Figure S1a) proved the high crystallinity of ZnO_2_, with all peaks assigned to the ZnO_2_ phase (PDF NO.13-0311), implying the desirable synthesis of pH-sensitive ZnO_2_ NPs. In addition, the acid-triggered properties of Zn^2+^ release and H_2_O_2_ production were investigated. Because of their pH-dependent destruction, the ZnO_2_ NPs released 95.5% and 7.5% of Zn^2+^ after incubation at pH 5 and 7.4 for 24 h, respectively, as measured by ICP–OES (Additional file 1: Figure S1b). Similarly, a large amount of H_2_O_2_ was generated with the acidic decomposition of ZnO_2_ (pH = 5), whereas little H_2_O_2_ was detected under an alkalescent environment (pH = 7.4) (Additional file 1: Fig.S1c). Collectively, these results indicated the effective pH-responsive release of Zn^2+^ ions and H_2_O_2_ from ZnO_2_ NPs.

ZnO_2_ NPs were then encapsulated in a mannose-decorated lipid bilayer (liposome, lipo) with catalase (Cat) to form functional ZnCM NPs [32]. As shown in Fig. 1b, monodispersed ZnO_2_, ZnC and ZnCM NPs with uniform morphologies were successfully synthesized, as evidenced by the evident membrane structures after encapsulation onto ZnO_2_ [33]. The zeta potentials of ZnO_2_, ZnC and ZnCM NPs were also determined to examine the changes in the NP surface characteristics. After lipid coating, the zeta potential changed from + 2.77 mV for ZnO_2_ to − 9.66 mV (ZnC) and then to − 14.1 mV (ZnCM NPs) (Fig. 1c), indicating successful lipid encapsulation. Herein, the increased negative surface potential of ZnCM compared with ZnC could be attributed to the proton dissociation of the targeting mannose moiety, which implied the successful decoration.

Previous studies have shown that ZnO_2_-based nanoplatforms could enhance oxidative damage in cancer cells by increasing mitochondrial H_2_O_2_ and releasing endogenous reactive oxygen species (ROS), thereby promoting cell death [32, 34–36]. Therefore, it appeared that detrimental H_2_O_2_ could directly destroy DCs instead of inducing immune-tolerant tDCs. Hence, it became of great significance to decrease the cytotoxicity of ZnO_2_ NPs while maintaining their Zn^2+^-associated DC tolerogenic ability. It has been shown that DC-involved immune responses take place under oxygen-limited conditions [18], which reminds us that inducing H_2_O_2_ to generate O_2_ could not only impede the oxidative reactions of ROS but also alleviate the hypoxic state of the activated DCs, altogether contributing to the induction of tDCs. Therefore, we coencapsulated ZnO_2_ and Cat with liposomes to develop an acid-sensitive catalytic oxygen (O_2_)-producing cascade nanosystem (ZnCM NPs). To further confirm that Cat was encapsulated into the liposomes, an SDS–PAGE experiment was performed (Fig. 1d). By comparing free Cat with acidolysed ZnCM NPs, a single molecular weight protein blot was observed at the same location, which indicated the successful encapsulation of Cat into the nanosystem. To explore the acid-dependent O_2_ release from ZnC and ZnCM NPs, we determined the dissolved oxygen concentrations by immersing the NPs in different PBS solutions (pH = 7.4, pH = 5) using a portable dissolved oxygen meter. As shown in Fig. 1e, both ZnC and ZnCM NPs showed rapid O_2_ production immediately after immersion in an acidic solution (pH = 5). In contrast, negligible O_2_ was detected at pH 7.4. These results were consistent with the acid-triggered H_2_O_2_ production nature of the ZnO_2_ NPs. The Zn^2+^ release characteristics of ZnC and ZnCM NPs under different pH conditions were also studied (Fig. 1f). As expected, Zn^2+^ showed distinct acid-sensitive release properties from ZnC and ZnCM NPs. Collectively, these results showed that biofunctional Zn^2+^ and O_2_ could be released effectively in an acidic environment from an acid-sensitive catalytic ZnCM cascadic nanosystem.

### Biocompatibility of the ZnCM nanoparticles

**Fig. 2:**
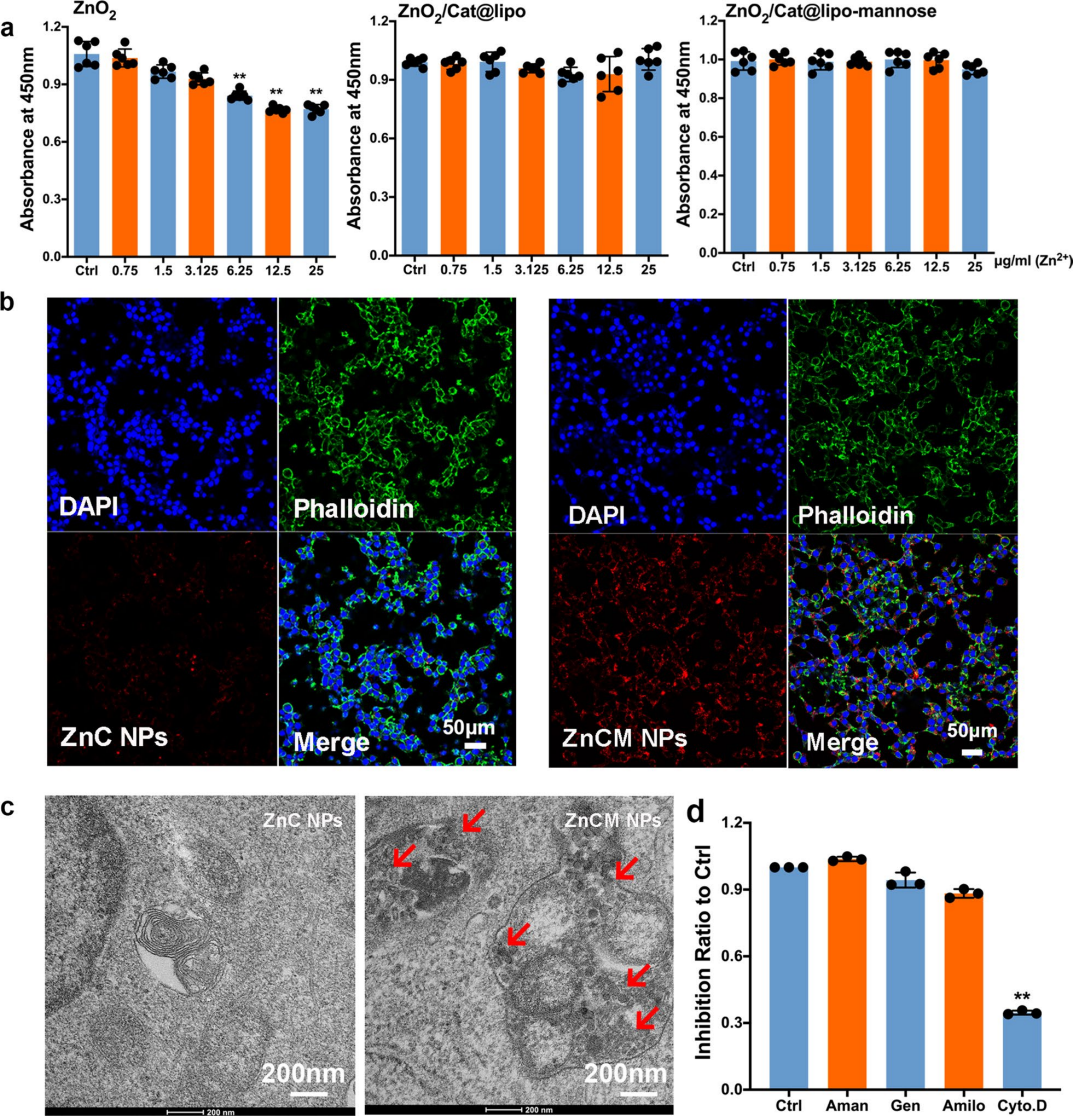
DCs targeting delivery of nontoxic ZnCM NPs in vitro. **a** Cell viabilities of DCs after varying NPs treatments. **b** In vitro CLSM observations of DCs treated with nontoxic ZnC (left) and ZnCM (right) NPs after 2 h. **c** In vitro Bio-TEM investigations of DCs treated with nontoxic ZnC and ZnCM NPs after 2 h. The red arrow indicated the intracellular localization of NPs in DCs. **d** Characterizations of route for NPs uptake by varying endocytosis inhibitors on DCs treated with nontoxic ZnCM NPs. ** indicated the significant difference of p ≤ 0.05 compared with Ctrl

Next, we investigated the biocompatibility of the various Zn^2+^ NPs (Fig. 2a). After ZnO_2_ NP treatment, the results showed that the DC viability decreased significantly with increasing ZnO_2_ concentrations, as evidenced by the minimal cytotoxicity at 6.25 μg/ml ZnO_2_ compared with the control (Ctrl), which was significantly different. In contrast, after coencapsulation with Cat and lipo, Zn^2+^ from ZnC NPs in the range of concentrations of 0.75 to 25 μg/ml barely showed significant cytotoxicity against DCs in vitro, indicating the noncytotoxicity of the modified NPs via the elimination of toxic H_2_O_2_ in vitro. After further loading with both Cat and mannose, the ZnCM NPs ranging from 0.75 to 25 μg/ml hardly exhibited any ability to kill DCs. These results demonstrated that ZnO_2_ NPs exerted oxidative damage in DCs, which could be alleviated by coencapsulation with Cat, implying the potential biocompatibility of ZnCM NPs in DC-targeted immune responses.

### Cellular uptake of the nanoparticles in vitro

DCs have been deemed to be vital during immune response initiation; therefore, targeted drug delivery into DCs not only presents efficient immune regulatory functionality but also, more importantly, avoids the undesirable leakage of ZnO_2_. Numerous DC-targeted ligands have been explored in previous DC-mediated immunotherapies, such as LSECtin, DC-SIGN [37], and mannose [26, 38]. Herein, mannose was further anchored onto our nanocarrier for targeted delivery into DCs. In Fig. 2b, after 2 h of incubation, we found that compared with the nontargeted ZnC NPs, fluorescent ZnCM NPs showed better intracellular localization within the DCs, indicating the DC-targeting ability of ZnCM. Additional in vitro Bio-TEM observations confirmed the CLSM results, as evidenced by the significant ZnCM NP uptake in DC intracellular lysosomes (Fig. 2c), while few ZnC NPs were found in DC sections. Further CLSM observations confirmed these lysosome-colocalization results, showing the ZnCM NPs were uptaken by DC lysosomes (Additional file 1: Figure S2). These results showed that the ZnCM NPs specifically entered DC lysosomes in vitro, which enabled the pH-dependent release of Zn^2+^ and O_2_ during the immune response of ZnCM NPs.

Furthermore, we aimed to unravel the underlying internalization mechanisms of ZnCM NPs into DCs. Several possible routes were found to participate in the uptake of the exogeneous NPs, such as clathrin-mediated endocytosis, caveolin-mediated endocytosis, micropinocytosis and clathrin-caveolin-independent endocytosis [39]. Hence, various pharmacological inhibitors (amantadine, genistein, amiloride and cytochalasin D) were used. Amantadine prevented the budding of clathrin-coated pits; genistein retarded the activation of the Src family of tyrosine kinases; amiloride inactivated Na^+^/H^+^ channels; and cytochalasin D destroyed the cytoskeleton to inhibit NP transportation [40]. Figure 2d showed that cytochalasin D significantly suppressed the internalization efficiency of ZnCM NPs compared to amantadine, genistein and amiloride after 2 h of incubation, indicating the feasible participation of the DC cytoskeleton in the process of ZnCM NP ingestion, which was independent of clathrin-caveolin-mediated endocytosis.

### Intracellular pH-dependent Zn^2+^ and O_2_ release from the nanoparticles

Acidic lysosome-dependent uptake of external stimuli was found to play vital roles in the biological behaviour of DCs [41, 42]. Herein, ZnCM NPs were taken up by DC lysosomes in a manner that was dependent on the cellular cytoskeleton transition. NPs characterization showed efficient pH-dependent Zn^2+^ and O_2_ extracellular release from the ZnCM NPs in vitro. Therefore, the intracellular Zn^2+^ and O_2_ release of ZnCM NPs in DCs was investigated.

**Fig. 3:**
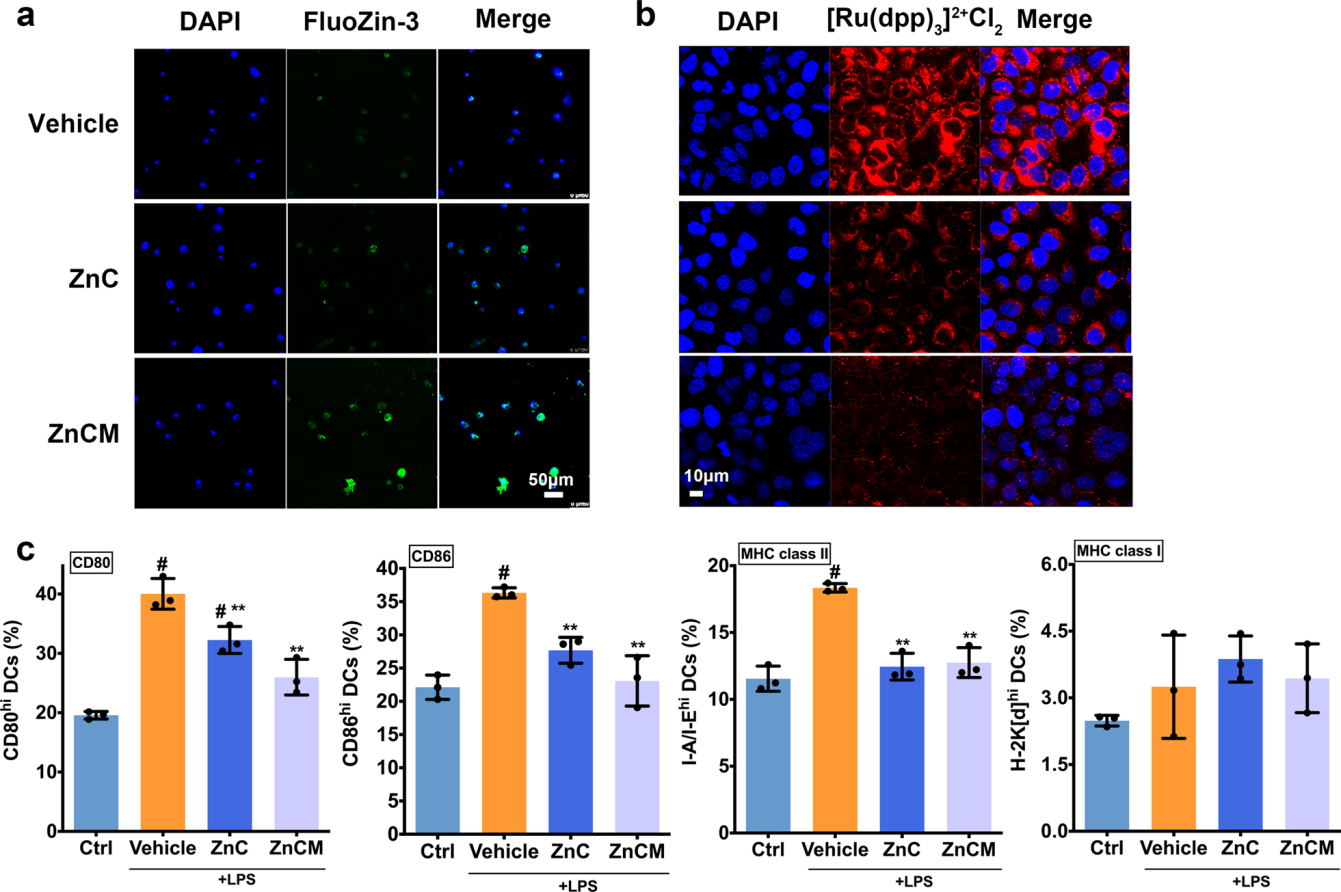
Zn^2+^ and O_2_ homeostasis regulation induced tDCs transition by ZnCM NPs. **a** CLSM observations of intracellular Zn^2+^ in DCs after various NPs treatments. **b** CLSM observations of intracellular hypoxia in DCs after various NPs treatments. **c** Quantitative flow cytometry of igDCs molecules (signal 1,2) after ZnC and ZnCM NPs treatments. ** indicated the significant difference of p ≤ 0.05 compared with Vehicle, # indicated the significant difference of p ≤ 0.05 compared with Ctrl

Treatment with Zn^2+^ ions has attracted great attention in comparison with other metal ions due to its distinct therapeutic characteristics [43]. Various biological metabolic pathways have been found to include zinc homeostasis, such as enzymatic activity, DNA transcription, protein synthesis and vascularization [44]. Zinc oxide (ZnO) NPs have been widely acknowledged as biocompatible candidates for desirable applications in tissue repair, antibacterial, anti-inflammatory, biomedical imaging and nanocarrier development [45, 46]. Nonetheless, the performance of ZnO was significantly restrained owing to its crystalline stability, leading to a lower level of active Zn^2+^. Therefore, we synthesized ZnO_2_-based ZnCM nanoclusters via a green, portable, and convenient process that not only released Zn^2+^ effectively to increase DC intracellular Zn^2+^ levels for immune response suppression but also further decomposed the toxic byproduct H_2_O_2_ into O_2_, which ingeniously alleviated the hypoxic state of activated DCs. Figure 3a demonstrated that compared with ZnC-treated igDCs, ZnCM NPs effectively released zinc ions in igDCs, as exemplified by the increased CLSM signals, which indicated that the intracellular Zn^2+^ concentration was significantly increased after biocompatible ZnCM NP administration. Additionally, the O_2_ levels in DCs were augmented significantly after NP treatment, as shown by the decreased fluorescence of the O_2_ indicator [Ru(dpp)_3_]^2+^Cl_2_, which was used to detect the hypoxic state [33] (Fig. 3b, Additional file 1: Figure S3). Notably, the ZnC NPs showed reduced red fluorescence in DCs in comparison with the vehicle, signifying the intracellular decomposition of H_2_O_2_ from the NPs catalysed by Cat. More importantly, the [Ru(dpp)_3_]^2+^Cl_2_ fluorescence in the DCs was alleviated more significantly after treatment with ZnCM NPs than ZnC NPs, indicating that targeted delivery into DCs via mannose was able to ameliorate hypoxia effectively. Hence, these results indicated that compared with vehicle and ZnC, ZnCM NPs could release Zn^2+^ and O_2_ efficiently within the targeted DCs in a pH-dependent manner, which encouraged us to explore the subsequent immune effects of these NPs to induce tDCs from igDCs.

### In vitro induction of tDCs by nanoparticles

During immune activity, DCs express pattern recognition receptors (PRRs) in response to extracellular signals, which is followed by a series of phenotypic and functional transformations termed the DC activation phase. These activities lead activated DCs to differentiate into immunogenic APCs, propelling the downstream proliferation and differentiation of antigen-specific CD4^+^ T cells into their corresponding effector cells [47] to initiate immune activity. The surface molecules presented on DCs are normally major histocompatibility complex (MHC) markers, which deliver the first-line activating stimulus to T cells and are therefore referred to as “signal 1”. In addition, a variety of costimulatory molecules, such as CD80 and CD86, are engaged in mediating signals that are vital for T-cell fate, known as “signal 2”. Finally, igDCs release a number of extracellular mediators (“signal 3”) to stimulate downstream CD4^+^ T cells, such as IL-1, IL-6, IL-12α, IL-12β, and TNF-α, to initiate the development of the T-cell response, altogether contributing to the origination of the activated adaptive immune response in RA [48]. Hence, after elucidating the intracellular Zn^2+^ and O_2_ release from the ZnCM NPs in DCs, we sought to uncover the potential NP immune induction of tolerogenicity in DCs by exploring the possible repression of signals 1–3.

To investigate the role of ZnCM NPs in the regulation of zinc ion homeostasis towards DC tolerogenicity, we first explored the expression of surface markers (signals 1 and 2) on igDCs via flow cytometry analysis (Fig. 3c). Upon stimulation with LPS, igDCs (vehicle) exhibited higher expression of CD86, CD80 and MHC class II on CD11c + cells, which indicated the activation of igDCs. However, after administration of both ZnC and ZnCM NPs, the DC levels of CD86, CD80 and MHC class II (signals 1 and 2) decreased significantly compared with the vehicle group, indicating the suppressed immunogenicity of DCs after zinc supplementation. Further immunofluorescent staining confirmed the above findings (Additional file 1: Figure S4), as demonstrated by the decrease in signal 1- and signal 2-positive staining of the DC maturation surface markers after Zn^2+^ NP treatments compared with Vehicle treatment. All these data showed that ZnO_2_-based NPs could switch igDCs towards tDCs, which was attributed to the increased intracellular Zn^2+^ and O_2_ concentrations.

Upon LPS stimulation of surface ligands, DCs activate NF-κB phosphorylation to promote targeted mRNA transcription, including IL-1, IL-6, IL-12α, IL-12β, and TNF-α (signal 3), thereby triggering a subsequent immune response [49]. Herein, we found that after stimulation with LPS, igDCs were activated, as demonstrated by the increased mRNA levels of IL-1, IL-6, IL-12α, IL-12β, and TNF-α compared with the those of the Ctrl. However, ZnC NPs decreased the mRNA levels of IL-1, IL-6, and IL-12β, while ZnCM NPs significantly inhibited those of IL-1, IL-12β, and TNF-α in contrast with LPS-induced vehicle (Additional file 1: Figure S5a). Cytometric bead array (CBA) immunoassays also showed that igDCs could produce larger amounts of IL-6, IL-12, and TNF-α than Ctrl cells, which were reduced significantly in response to ZnC and ZnCM NP administration (Additional file 1: Figure S5b). These data indicated that ZnCM NPs potently suppressed LPS-induced DC immunogenicity by repressing signals 1, 2 and 3, implying the potential immune tolerogenicity of ZnCM NPs in vitro.

### Repression of T cells by tDCs after nanoparticle treatment

The interaction between DCs and CD4^+^ T cells was shown by an increase in a series of key biomarkers, such as RANKL, IL-12, IFN-γ, and TGF-β [50], which promoted the development and progression of RA. igDC stimulation activated CD4^+^ T-cell homeostasis by facilitating the release of inflammatory cytokines from functional T cells [51]. Therefore, we analysed the proliferation of CD4^+^ T cells and their CBA levels in vitro after coculture with NP-treated igDCs to identify the role of ZnCM NPs in the modulation of tDC capability towards T cells [49, 52]. First, the proliferation of splenic OT-II CD4^+^ T cells was assessed by flow cytometry, which showed that coculture with LPS-treated DCs (igDCs) promoted the proliferation of CD4 + T cells. However, ZnC/ZnCM NP-treated cocultured tDCs had a reduction in the numbers of CD4 + T cells and their proliferation compared with those of LPS-treated igDCs, indicating that both ZnC and ZnCM NPs induced the ability of tDCs to silence T cells (Additional file 1: Figure S6a). Furthermore, CBA analysis of CD4^+^ T cells after DC coculture demonstrated that despite the increase in IL-17 and IFN-γ in T cells after LPS-treated igDC coculture, both ZnC and ZnCM NPs decreased this tendency, among which ZnCM showed more significantly decreased levels of IL-17 and IFN-γ than ZnC NPs (Additional file 1: Figure S6b). Therefore, these results suggested that ZnCM NPs could affect the proliferation and activation of CD4^+^ T cells by modulating the immune crosstalk between tDCs and functional CD4^+^ T cells.

### Repression of the deubiquitinase OTUB1 induced tDCs by CCL5 degradation via NF-κB signalling

**Fig. 4:**
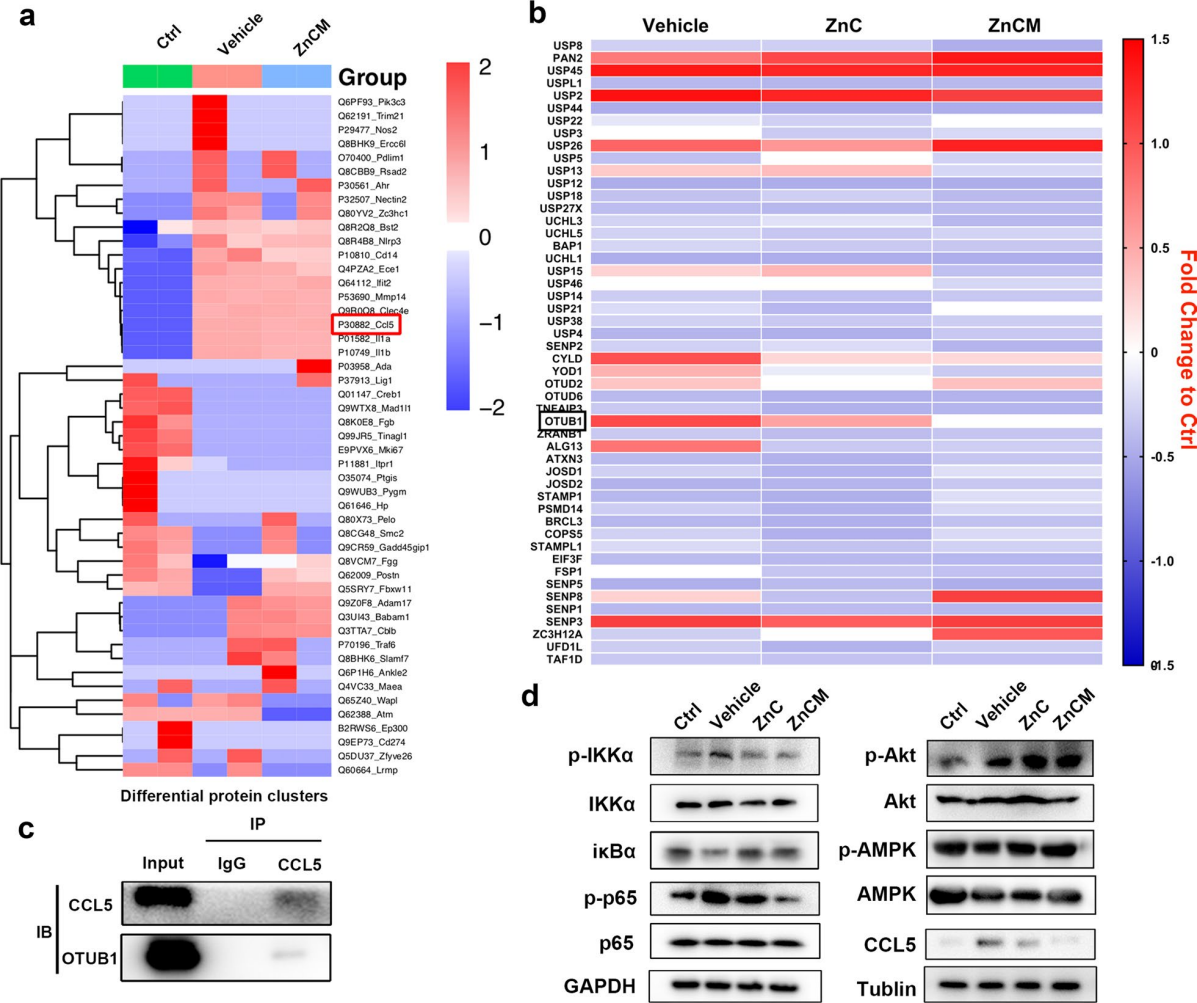
ZnCM NPs induced tDCs transition to inhibit CD4^+^ T cell homeostasis via OTUB1/CCL5/NF-κB. **a** Label-free quantitative proteomics showed the targeted protein molecules in igDCs affected by NPs. **b** Quantitative real-time PCR analyzing ubiquitination and deubiquitination proteasome systems of igDCs treated with NPs. **c** Co-Immunoprecipitation analysis investigated the binding ability of OTUB1 and CCL5. **d** Western blot analysis explored the regulative NF-κB, Akt and AMPK signaling in modulating CCL5 expression of igDCs treated with NPs

The data reported above suggested that ZnCM NP targeted delivery into DCs could induce tDC transition to suppress CD4^+^ T-cell activation, thereby inhibiting the progression of RA. To further explore the mechanism by which hyperoxic Zn^2+^ homeostasis regulates igDC generation, label-free quantitative proteomics analysis of igDCs treated with ZnCM NPs was performed with an RPLC–MS/MS system. Among the differentially expressed proteins in the various groups, protein intensity based on the MaxLFQ algorithm was used to analyse all the proteins in each sample, as shown by the normalized LFQ intensity ratio > 1 or < 1 between groups. The proteomics results showed that the numbers of upregulated proteins and downregulated proteins were 133 and 149 in the Vehicle vs. Ctrl groups, 154 and 154 in the ZnCM vs. Ctrl groups, and 124 and 106 in the ZnCM vs. Vehicle groups. Among all the positive protein candidates screened, the proteins IL-1α, IL-1β, CCL5, Rnaseh2c, Vwde, Cd14, and Bst2 showed the most significant vehicle/Ctrl ratios > 1, indicating their increased protein level in igDCs compared with Ctrl. More importantly, these candidates also exhibited a ZnCM/vehicle ratio ≤ 0.85, indicating significantly decreased protein levels in igDCs after ZnCM NP treatment (Fig. 4a). Herein, CCL5 [chemokine (C–C motif) ligand 5], which leads immune cell migration towards targeted inflammatory sites, was found to play an important role in regulating the local immune response [53]. Moreover, CCL5 can mediate the recruitment of T cells and eosinophils [54], resulting in the activation of the T-cell response. Therefore, we selected CCL5 as the target molecule for Zn^2+^ homeostasis regulation against igDCs in RA. Our proteomics study suggested that ZnCM downregulated the expression of CCL5 in igDCs, inducing the activation of tDCs to further inhibit the T-cell response.

Next, we sought to identify the mechanisms regulating the decreased level of CCL5 in tDC transformation by ZnCM NPs. The ubiquitin–proteasome system (UPS) is an indispensable regulatory system for cellular protein homeostasis that balances intracellular protein degradation [55]. Particularly in DCs, the suppression of deubiquitinase function in the UPS cascade leads to alterations in DC phenotype and function, which has propelled the development of novel immunotherapies [56–58]. Based on these findings, we screened a series of ubiquitinases and deubiquitinases in igDCs treated with NPs. Figure 4b revealed several active candidates that regulated protein degradation, among which the OTU domain of ubiquitin aldehyde binding 1 (OTUB1) showed a significant decrease in ZnCM-treated igDCs compared with vehicle-treated igDCs. Previously, OTUB1 had emerged as an effective deubiquitinase to cleave K48-linked ubiquitin chains to inhibit protein degradation [59]. It was reported that OTUB1 deubiquitination is closely associated with the biological functions of DCs, which inhibit CD4^+^ T-cell proliferation and IFN-γ cytokine secretion in allogenic mixed lymphocyte reactions (allo-MLRs) [59]. Our results suggested that ZnCM NPs significantly downregulated the expression of the deubiquitinase OTUB1 in igDCs. Therefore, we selected OTUB1 as the target kinase for Zn^2+^ homeostasis regulation by ZnCM NPs to induce tDCs in RA.

However, the possible crosstalk between CCL5 and OTUB1 has yet to be explored. Hence, immunoprecipitation (IP) analysis was employed and showed the intimate connection between OTUB1 and CCL5 in DCs (Fig. 4c), indicating that CCL5 could serve as the target molecule for OTUB1 deubiquitination. Specifically, by inhibiting the activation of OTUB1 deubiquitination, ZnCM NPs could promote CCL5 degradation to induce tDCs and further inhibit the T-cell response, exhibiting Zn^2+^ homeostasis regulation by ZnCM NPs in RA.

In addition, it was shown that the expression of CCL5 was closely related to the activation of NF-κB signalling, leading to regulatory effects on cell fate [60]. Previous studies have shown that extracellular stimuli can activate NF-κB signalling, which contributes to the increased expression of CCL5 [61]. In addition, OTUB1 was found to modulate Toll-like receptor (TLR)-induced igDC maturation via NF-κB signalling, as exemplified by activation of the NF-κB kinases TRAF6 and TAK-1 [62]. These studies implied the possible participation of NF-κB signalling in Zn^2+^ homeostasis regulation against RA. Herein, we initially verified the downregulated expression of CCL5 in NP-treated DCs compared with vehicle-treated igDCs (Fig. 4d). Next, we found that compared to vehicle-treated igDCs, ZnCM-treated igDCs showed decreased expression of p-IKKα and p-p65 and increased levels of iκBα, indicating inhibited activation of NF-κB signalling. Moreover, the Akt and AMPK pathways hardly showed significant effects after NP intervention in comparison with vehicle-treated igDCs, implicating that the Akt and AMPK pathways were not actively involved in mediating Zn^2+^ homeostasis regulation in RA (Additional file 1: Figure S7). Collectively, the data above demonstrated that ZnCM NPs could inhibit the activation of OTUB1 deubiquitination, thereby promoting CCL5 degradation via NF-κB signalling to induce tDCs for further T-cell response inhibition, which explained the molecular mechanisms of Zn^2+^ homeostasis regulation by ZnCM NPs to treat RA.

### Nanoparticle localization in vivo

**Fig. 5:**
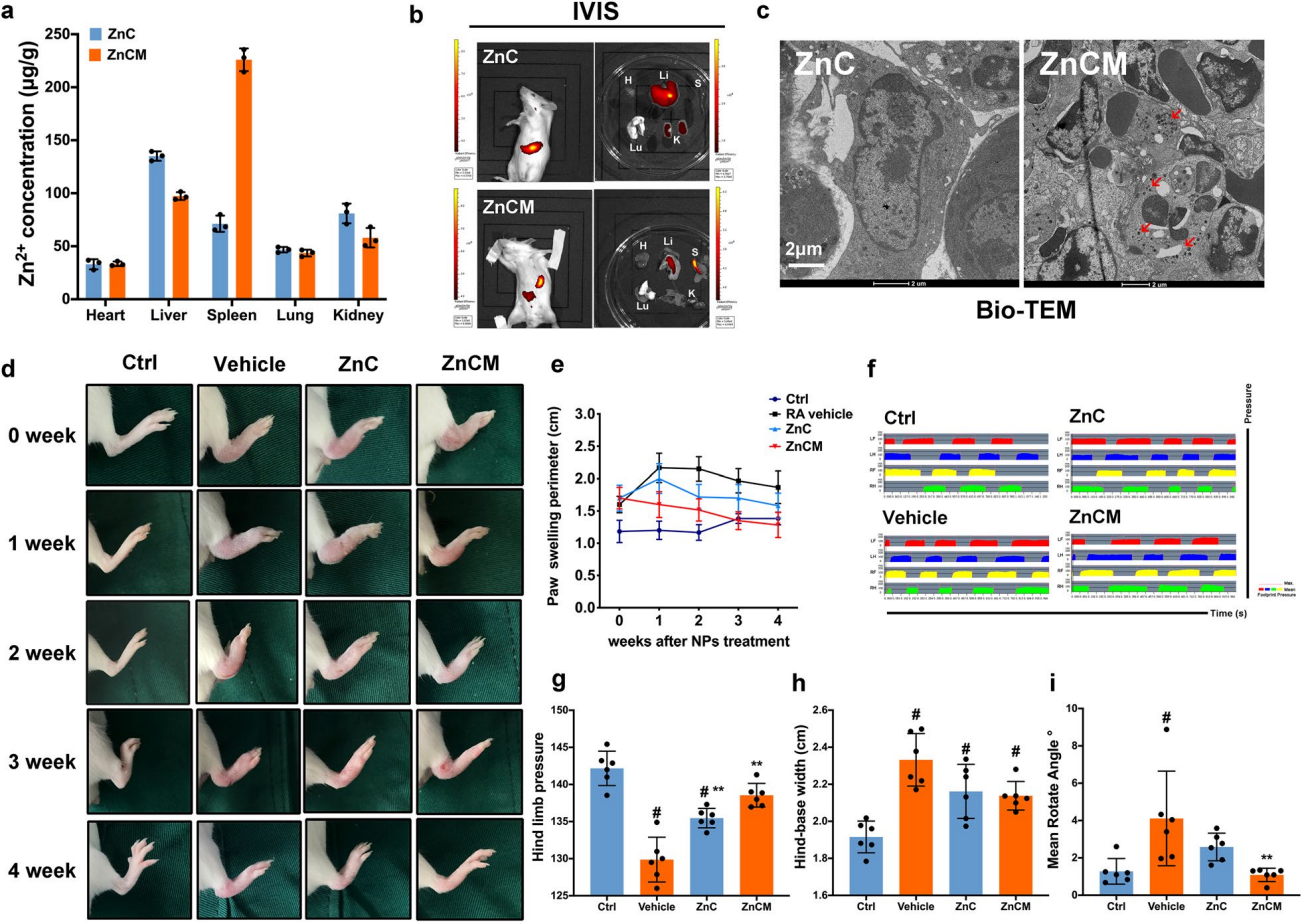
ZnCM NPs attenuated RA progression in vivo by targeting splenic DCs. **a** The bio-distribution pattern in vivo of ZnC, ZnCM NPs in disparate organs based on Zn^2+^ concentrations. **b** IVIS imaging of RA mice injected with ZnC, ZnCM NPs retrobular (H: Heart, Li: Liver, S: Spleen, Lu: Lung, K: Kidney). **c** In vivo Bio-TEM investigations of splenic DCs in RA mice treated with ZnC, ZnCM NPs. The red arrow indicated the in vivo localization of NPs in splenic DCs. **d** Alleviation of ankle swelling after NPs treatments in RA mice. **e** Quantitative measurements of swelling paw perimeters weekly. **f** Pressure–time diagram showed the time course within single stance highlighted by different colors for each limb in RA mice injected with NPs from visual gait analysis system. **g** Quantitative results of hind limb pressure in RA mice injected with NPs. **h** Quantitative results of hind-base width in RA mice injected with NPs. **i** Quantitative results of mean rotate angle in RA mice injected with NPs. LF: left front limb, RF: right front limb; LH: left hind limb, RH: right hind limb. ** indicated the significant difference of p ≤ 0.05 compared with Vehicle, # indicated the significant difference of p ≤ 0.05 compared with Ctrl

It is unanimously believed that the spleen is the largest immune organ of the whole body and accumulates the greatest number of immune cells. Hence, it is proposed that splenic DCs could serve as an ideal target for ZnCM localization in vivo. Herein, collagen-induced arthritis (CIA) mice representing an RA model were injected with various NPs. Additional file 1: Figure S8 showed the normal physiological morphologies of the heart, liver, spleen, lung, and kidney after CIA RA mice were injected with NPs for 4 weeks, indicating the in vivo biosafety of the ZnC and ZnCM NPs. Next, we sought to uncover the in vivo localization of the ZnCM NPs. As shown in Fig. 5a, the Zn^2+^ contents in 1 g of heart, liver, spleen, lung, and kidney tissue (µg/g) from ZnC- and ZnCM-treated mice were determined, and a higher concentration of Zn^2+^ was found in the spleen after ZnCM NP injection than in the liver, heart, lung, or kidney after ZnC NP injection. Furthermore, the ex vivo fluorescence data of the targeted organs from RA mice injected with ZnC NPs demonstrated that these NPs showed significantly more accumulation in the liver than in other organs (Fig. 5b). However, after administration of ZnCM NPs, the fluorescence signal in the spleen increased significantly, leading to a relative decrease in fluorescence intensity in the liver, indicating the possible spleen-targeting ability of ZnCM NPs in vivo. These results indicated that ZnCM NPs most significantly targeted the spleen in RA mice, thereby exerting tolerogenicity on DCs in vivo. This effect could be explained by the greater accumulation of immune cells in the spleen than in the liver and the effective DC-targeting effects of ZnCM NPs, which resulted in the localized splenic targeting of ZnCM NPs in vivo. Next, we used Bio-TEM to observe the intracellular localization of the NPs in the spleen in vivo. Compared with ZnC NPs, ZnCM NPs were internalized in the lysosomes of splenic DCs (Fig. 5c). The above in vivo data indicate that in RA mice, ZnCM NPs significantly targeted DCs in the spleen in comparison with the targeting of ZnC NPs.

### Alleviation of rheumatoid arthritis manifestations in vivo by nanoparticles

After NP localization was confirmed in splenic DCs, the in vivo anti-RA effects of the ZnCM NPs were studied. Figure 5d and e show that 4 weeks after NP administration, compared to Ctrl mice, RA mice (vehicle-treated) developed significant swelling of the ankle in the hind ankle, which peaked at 1–2 weeks and was followed by a slight decline for the subsequent 2 weeks; this result indicated a certain amount of self-relief after the excessive immune response in RA CIA mice. In addition, after ZnC NP treatment, the RA mice developed an increase in the degree of swelling in the hind leg ankle at 1 week, which was followed by a significant decrease for the following week compared to vehicle-treated mice. However, no significant difference was found in ankle swelling between vehicle- and ZnC-treated mice in the 3rd and 4th weeks. In contrast, after ZnCM NP treatment, the hind limb ankle experienced a consistent and gradual reduction in the swelling perimeter for 4 weeks compared with RA mice, denoting the significant RA treatment efficacy of ZnCM NPs in vivo.

In addition, inflammation in the hind ankle can cause pain while walking, thus affecting walking gait posture. Therefore, we performed gait analysis to provide a quantitative assessment of behavioural walking changes in RA mice injected with various NPs. Walking gait data were first collected by recording the footprints on the sensor screen. It was found that compared with Ctrl mice, RA mice tended to shift their body weight to the front and avoid stepping on their inflamed hind limbs, resulting in reduced pressure from the hind pawprints compared with the front pawprints. However, after effective treatments, prediseased mice induced an even, balanced pressure on all four paws. Figure 5f showed the changes over the time course of the walking single-limb stance time in the different treatment groups. The RA mice had a reduction in the single time stance of the hind limbs (shown in blue and green), which was significantly recovered by treatment with ZnCM NPs. The quantitative hind limb pressure results indicated that the hind paw pressure of both the ZnC- and ZnCM NP-treated mice increased significantly compared to that of RA mice, despite the evident decrease in hind paw pressure in RA mice compared with Ctrl mice (Fig. 5g). These results indicated that ZnCM NP treatment ameliorated the changes in walking posture caused by RA, which might be attributed to the alleviation of inflammatory pain by NP treatment. Furthermore, the hind-base width, which represents the average distance between the two hind paws, was analysed to evaluate the coordination of RA mice (Fig. 5h). Diseased mice were inclined to place their hind paws farther apart than Ctrl mice, which has been labelled the hallmark of unsteady gaits [63]. After treatment with ZnC and ZnCM NPs, the hind-base widths were partially restored compared with those of RA mice, indicating that the ZnCM NPs could ameliorate walking imbalance to a certain extent compared to RA mice. The mean rotation angle is the average value of the angle between the axis from the mouse mouth tip to the tail tip along the central longitudinal axis. This angle represents a twisted posture of the mouse body while walking caused by the inflamed ankle [64]. Figure 5i demonstrates that there was an increase in the mean rotation angle in RA mice compared with Ctrl mice, which was attributed to severe inflammation in the hind ankle. In contrast, compared with RA mice, the mean rotation angle decreased significantly after ZnCM NP treatment, indicating the efficient alleviation of RA ankle inflammation. In summary, ZnCM NP treatment improved the pathological walking patterns of RA mice in terms of hind limb pressure, mean rotation angle and stance time course, thereby suggesting the alleviation of RA manifestations in vivo and indicating the potential clinical efficacy of ZnCM NPs for RA treatment.

### Effects of the nanoparticles on osteolysis and inflammation in vivo

**Fig. 6:**
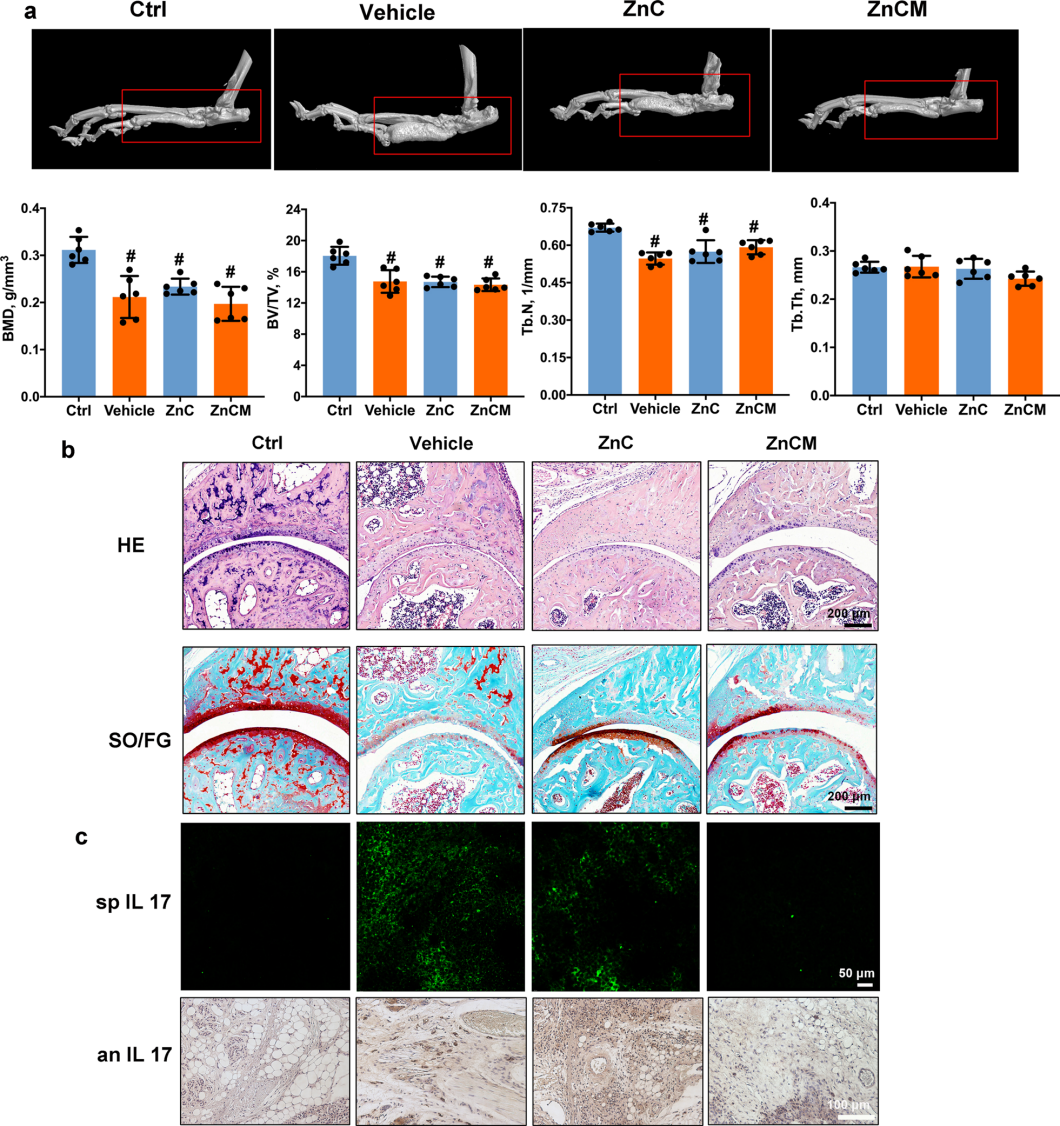
Effects on osteolysis, joint cartilage and inflammation in vivo after RA mice injected with NPs. **a** μCT results of ankle joint in RA mice injected with NPs for 4 weeks. **b** HE and safranin O/ fast green (SO/FG) staining of ankle joint in RA mice injected with NPs for 4 weeks. **c** Inflammatory IL-17 staining of spleen (sp) and ankle joint (an) in RA mice injected with NPs for 4 weeks. ** indicated the significant difference of p ≤ 0.05 compared with Vehicle, # indicated the significant difference of p ≤ 0.05 compared with Ctrl

Bone destruction and cartilage damage have emerged as the key milestones during RA progression and indicate its severity and prognosis [65, 66]. Therefore, the diseased ankles of RA mice after NP injection were analysed by μCT (Fig. 6a). Quantitative results showed that RA decreased the Tb.N, BV/TV, and BMD compared with those of Ctrl mice, indicating significant bone destruction caused by RA. However, both ZnC and ZnCM NP treatments failed to protect bone destruction after 4 weeks of administration, as exemplified by the Tb.Th, Tb.N, BV/TV, and BMD data that hardly exhibited significant differences compared with those of RA mice. These results indicated that although ZnCM NPs could alleviate RA immune inflammation, they affected limited osteoclast-mediated osteolysis in vivo after 4 weeks of treatment. Thus, longer treatment and observation times may be needed.

Furthermore, HE and safranin O/fast green (SO/FG) staining (Fig. 6b) showed that RA mice had significant cartilage erosion and synovial inflammation compared with Ctrl mice. Both ZnC and ZnCM NPs not only protected against cartilage damage but also inhibited synovial inflammation, and the ZnCM NPs exhibited a more significant effect than ZnC NPs. IL-17 (signal 3), a crucial cytokine secreted from immune cells that reflects the local inflammatory response, was also observed in stained sections from the ankle and spleen. Figure 6c showed that in RA mice, the levels of IL-17 were elevated in both the ankle and spleen, which was attenuated by ZnCM NP treatment, indicating that ZnCM NPs significantly inhibited inflammatory activity in both the ankle and spleen. Collectively, these data from the RA model mice indicated that ZnCM NPs were capable of inhibiting cartilage erosion and inflammation in the ankle and spleen despite the insignificant effects on osteolysis after 4 weeks of treatment.

### In vivo induction of tDCs by nanoparticles

**Fig. 7:**
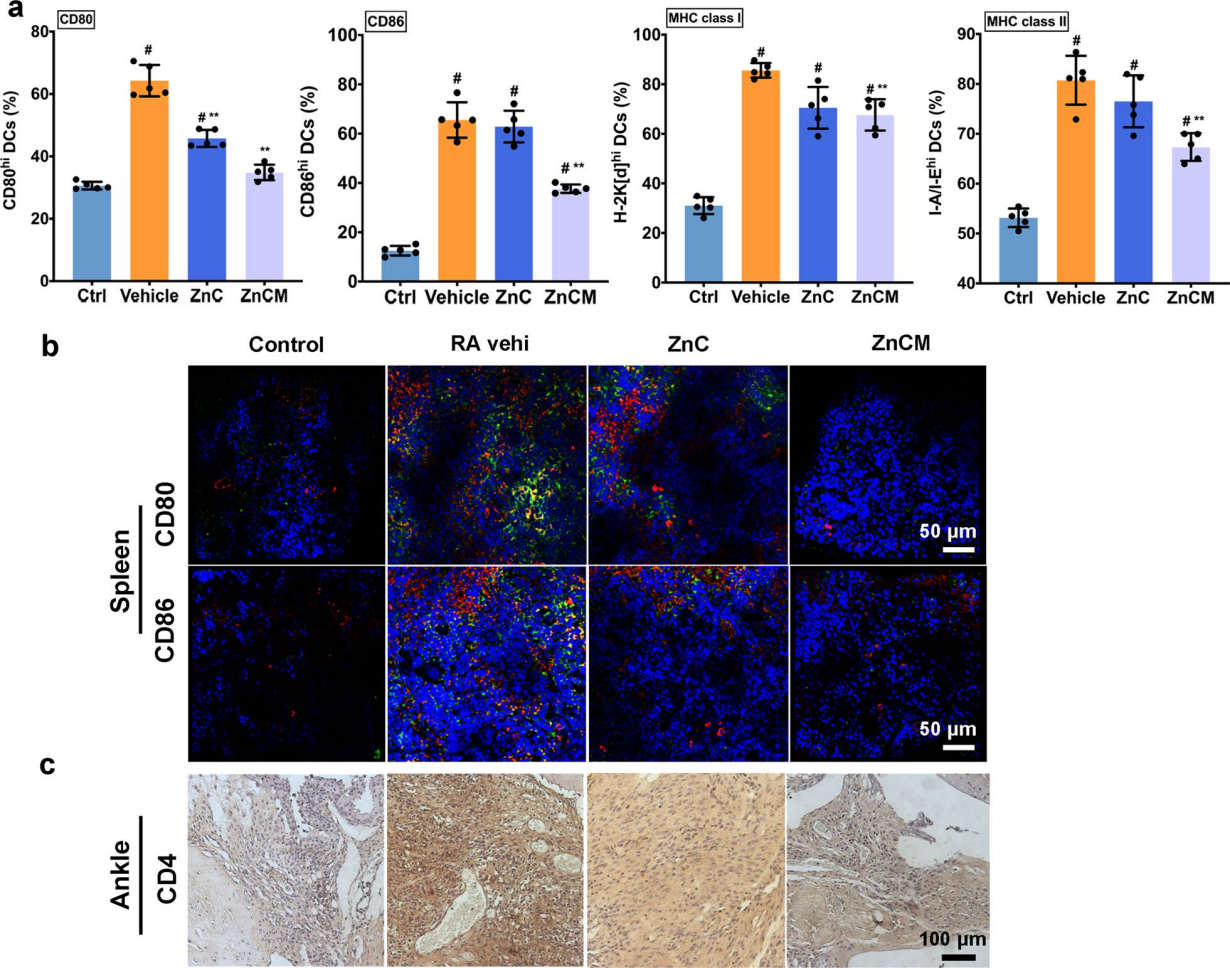
In vivo splenic tDCs induction by ZnCM NPs. **a** Quantitative flow cytometry of in vivo splenic DCs molecules after ZnC and ZnCM NPs treatments. **b** Observations of in vivo DCs CD80, CD86 expressions in spleen and **c** CD4^+^ T cells expression in ankle synovium after various NPs treatments. (blue: DAPI; red: CD11^+^ DCs; green: CD80/CD86 DCs; yellow: co-localization of CD11^+^ and CD80/CD86). ** indicated the significant difference of p ≤ 0.05 compared with Vehicle, # indicated the significant difference of p ≤ 0.05 compared with Ctrl

**Fig. 8:**
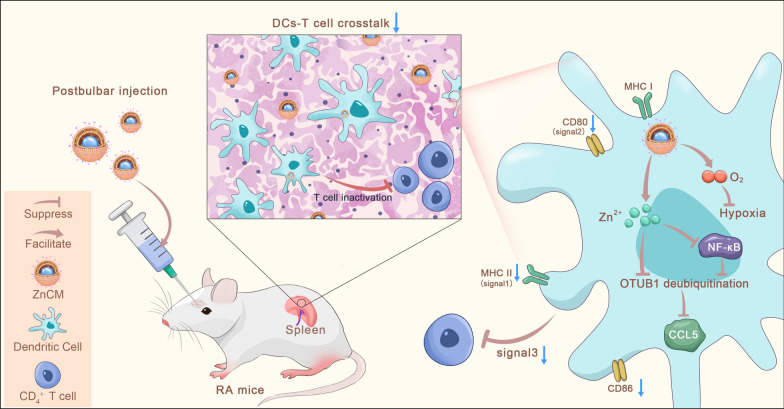
Schematic illustrations of immune-regulating strategy against rheumatoid arthritis by inducing tolerogenic dendritic cells with modified zinc peroxide nanoparticles

Next, we aimed to examine the effects of ZnCM NPs on DC tolerogenicity in vivo. After mice were administered the appropriate treatments, CD11c^+^ DCs from the spleen were harvested for flow cytometry analysis. Figure 7a showed that RA mice developed a significant increase in the expression of CD86, CD80, MHC class I and MHC class II in their CD11c^+^ DCs compared with expression in Ctrl mice. However, treatment with ZnC NPs reduced the level of CD80, while ZnCM NP treatment decreased the expression of CD80, CD86, MHC class I and MHC class II (signals 1 and 2). This result was consistent with those from CD molecule staining in vivo (Fig. 7b), demonstrating that the number of CD80^+^ and CD86^+^ CD11c^+^ DCs increased significantly after RA establishment but decreased after ZnCM NP treatment, indicating the suppressed immunogenicity of DCs after Zn^2+^ and O_2_ supplementation in vivo. Furthermore, Fig. 7c showed that the ZnCM NPs significantly affected the staining of CD4^+^ T cells in the ankle synovium compared with that of RA mice, indicating that ZnCM NPs effectively suppressed the immune crosstalk between tDCs and T cells in vivo to impede the progression of RA-mediated inflammation. All these data showed that modified ZnO_2_-based NPs could switch igDCs towards tDCs in vivo, indicating that Zn^2+^ homeostasis controlled by ZnCM NPs effectively regulated RA in vivo (Fig. 8).

## Conclusions

Tolerogenic DCs represent an innovative target for effective RA treatment. Herein, a DC-targeting ligand mannose (man)-anchored liposome coencapsulating ZnO_2_ and catalase (Cat) (ZnCM NPs) was developed to regulate intracellular zinc and oxygen homeostasis to induce tolerogenic DCs in RA. ZnCM NPs were shown to endow targeted intracellular delivery of Zn^2+^ and O_2_ towards DCs in a pH-responsive manner, thereby facilitating the transition from igDCs towards tDCs. Due to the inactivation of OTUB1 deubiquitination, ZnCM NPs promoted CCL5 degradation via NF-κB signalling to induce tDCs for further T-cell response inhibition, resulting in a repressed interaction between splenic CD11^+^ DCs and CD4^+^ T cells to inhibit RA progression. In vivo, this was shown by alleviated ankle swelling, improved walking posture, and inhibited ankle/spleen inflammation with disturbed activation of igDCs. Our work highlights the significance of zinc and oxygen homeostasis in treating RA by inducing tDCs with modified ZnO_2_ NPs, which provides novel insight into ion homeostasis regulation for cancer or immune disease therapy with a larger variety of distinct metal or nonmetal ions.

## Methods

### Reagents, cells, and antibodies

Zinc acetate (Zn(OAc)_2_), polyvinylpyrrolidone (PVP; MW 10,000), hydrogen peroxide (H_2_O_2_; 30 wt. % in H_2_O), and catalase (Cat; from bovine liver) were purchased from Sigma–Aldrich. Dipalmitoyl phosphatidylcholine (DPPC) and 1,2-distearoyl-sn-glycero-3-phosphoethanolamine-polyethylene glycol (DSPE-PEG; MW 2,000) were purchased from Ponsure Biotechnology Co., Ltd. (Shanghai, China), and 1,2-distearoyl-sn-glycero-3-phosphoethanolamine-polyethylene glycol-mannose (DSPE-PEG-M; MW 2,000) was purchased from Ruixi Biological Technology Co., Ltd. (Xi’an, China). RPMI-1640 medium (12,633,012), foetal bovine serum (10,099), protease inhibitor cocktail (78,438), an oxygen probe (44,123–02), Tris(4,7-diphenyl-1,10-phenanthroline) ruthenium(II) dichloride, FluoZin-3 AM (2,145,045), CFSE (carboxyfluorescein diacetate succinimidyl ester; C34554) and an antibody for USP2 (PA5-48,091) were obtained from Thermo Fisher Scientific, USA. Magnetic beads coated with antibodies against mouse CD11c (130–125-835) and CD4 were obtained from Miltenyi Biotech. Lipopolysaccharide (LPS) (70-CS0006) was purchased from Lianke Biotech. The TruStain FcX (anti-mouse CD16/32, 422,302) antibody was purchased from BioLegend. RIPA buffer (P0013C) and a BCA protein assay kit (P0012S) were purchased from Beyotime Biotechnology. Recombinant mouse GM-CSF (granulocyte–macrophage colony-stimulating factor; 31,503) and IL-4 (21,414) were purchased from PeproTech. Conjugated antibodies against mouse CD80 (16-10A1, 560,016), I-A/I-E (2G9, 553,623), CD86 (GL-1, 560,582), H-2 k[d] (SF1-1.1, 553,566), CD11c (HL3, 562,782), and their corresponding isotype controls were obtained from BD Pharmingen. Antibodies against mouse IL-17 (ab79056), CD4 (ab183685), CD86 (ab112490), CD31 (ab28364), and CD80 (ab215116) were purchased from Abcam. Antibodies against CCL5 (2989), pIKKα phosphorylated at Ser176 and Ser180 (2697), pIkbα phosphorylated at Ser32 (2859), pp65 phosphorylated at Ser536 (3031), pAkt phosphorylated at Ser473 (4060), pAMPK phosphorylated at Thr172 (50,081), IKKα (61,294), Iκbα (4814), p65 (8242), Akt (9272), AMPK (5832), GAPDH (5174), tubulin (5666), and IgG (2729) were purchased from Cell Signaling Technology. TRIzol reagents were purchased from Invitrogen. The PrimeScript RT–PCR kit and SYBR Premix ExTaq kit were from Takara Bio. Type II collagen (20,021), CFA (7023), and IFA (7002) were purchased from Chondrex.

### Cell isolation and culture

For BMDCs, bone marrow cells were expelled from the femurs and tibiae of BALB/c mice and cultured with recombinant mouse GM-CSF (10 ng/mL) and IL-4 (1 ng/mL) as previously described [67]. On Day 6, CD11c^+^ BMDCs were isolated by positive selection with anti-mouse CD11c magnetic beads and collected as immature DCs (imDCs). imDCs were further cultured with LPS (100 ng/mL) for 24 h to generate mature igDCs. Splenic OT-II CD4^+^ T cells and CD11c^+^ DCs were enriched from splenocytes via positive selection with the corresponding magnetic beads. BMDCs and DC2.4 cells (SCC142, Sigma–Aldrich) were cultured in RPMI-1640 supplemented with 10% FBS.

### Synthesis of ZnO_2_ nanoparticles

One gram of Zn(OAc)_2_ and 1 g of PVP (MW 10,000) were dissolved in 50 mL of deionized water. Then, 5 mL of H_2_O_2_ (30 wt.%) was quickly added to the above solution with vigorous stirring. The colourless solution gradually turned milky white [32]. After reaction for 24 h, the ZnO_2_ NPs were obtained by high-speed centrifugation (13,000 rpm, 10 min) and washed with absolute ethyl alcohol three times. The resulting ZnO_2_ NPs were redispersed in absolute ethyl alcohol for long-term storage.

### Preparation of ZnC (ZnO_2_/Cat@lipo) and ZnCM (ZnO_2_/Cat@lipo-mannose) nanoparticles

A dried lipid film was prepared by following previous protocols [68]. In brief, a lipid mixture of DPPC, cholesterol, DSPE-mPEG (MW 2,000), and DSPE-mPEG-mannose (absent for the ZnC NP preparation) was dissolved and dried with a rotary evaporator. Next, the dried lipid film was hydrated with a mixed solution of Cat (1 mg/mL) and ZnO_2_ NPs (2 mg/mL). The lipid solution was stirred at 37 °C for 30 min, followed by extrusion through a 200-nm polycarbonate filter at 37 °C 11 times. The unencapsulated Cat and ZnO_2_ were removed with a Sephadex G-100 column. The obtained ZnCM NPs were condensed with an Amico filter device with a molecular weight cut-off (MWCO) of 100 kDa (Millipore, Bedford, MA) for further use.

### Nanoparticle characterizations

The morphological characteristics of the NPs were observed using an FEI Talos L120C transmission electron microscope (120 kV). The X-ray diffraction (XRD) patterns of the ZnO_2_ NPs were measured on a Rigaku D/MAX-2250 V device. The zeta potentials of the ZnO_2_, ZnC and ZnCM NPs were measured with Malvern Zetasizer equipment.

### In vitro Zn^2+^, H_2_O_2_ and O_2_ release

In vitro measurements of pH-triggered Zn^2+^ and H_2_O_2_ release from NPs were carried out by immersing the NPs in different PBS solutions (pH = 7.4, 5). The solutions were centrifuged to collect the supernatants at different time points. The concentration of Zn^2+^ in the supernatant was measured by ICP–OES (Agilent 700 Series), and the amount of H_2_O_2_ outside the dialysis tube was detected using a hydrogen peroxide assay kit (Beyotime Biotechnology Co., Ltd., China).

Then, a portable dissolved oxygen meter (Rex, JPBJ-608, China) was employed to detect the dissolved O_2_ in the aqueous solutions. The catalytic activities of the ZnC and ZnCM NPs were measured under the following four experimental conditions: a. ZnC (1 mg/mL) immersed in PBS at pH 7.4; b. ZnC (1 mg/mL) immersed in PBS at pH 5; c. ZnCM (1 mg/mL) immersed in PBS at pH 5; and d. ZnCM (1 mg/mL) immersed in PBS at pH 7.4. An oxygen electrode probe was inserted to measure the O_2_ concentration.

### Catalase loading verification

Equal volumes of ZnCM NPs and free Cat (positive control, PC) were predissolved for subsequent centrifugation at 13,000×*g*. A mixture of supernatant and loading buffer was separated with 10% SDS–PAGE. The bands were stained with Coomassie blue staining solution.

### Dendritic cell viability

Murine dendritic DC2.4 cells (7000 cells/well) were seeded and pretreated with 100 ng/ml LPS. Then, the igDCs were administered varying Zn^2+^ concentrations of ZnO_2_, ZnC, or ZnCM NPs for 24 h. Next, 100 μl of 10% CCK-8 solution (Dojindo, Japan) was added to each well for a 2-h incubation at 37 °C. DC viability was determined by measuring the absorbance at 450 nm with a microplate reader (Bio–Rad, USA).

### Nanoparticle uptake into dendritic cells

Nontoxic NPs containing 50 μg/mL Zn^2+^ were used for further experiments. Fluorescent ZnC and ZnCM NPs were added to igDCs (DCs treated with 100 ng/ml LPS) for 2 h. The endocytosis of Zn^2+^ NPs into the DCs was assessed after removal of the supernatant, followed by phalloidin and DAPI (4 ‘,6-diamidino-2-phenylindole dihydrochloride) staining of the cytoskeleton and nuclei. Confocal laser scanning microscopy (CLSM) (Leica TCS-SP5) was used to observe DC endocytosis of the NPs. We also investigated the intracellular location of the NPs in DCs via Bio-TEM (FEI, USA) and CLSM [69, 70]. Cells were seeded in 6-well plates and treated with nontoxic ZnC and ZnCM NPs. Next, DCs were harvested and fixed in 2.5% glutaraldehyde at room temperature for 1 h. Then, 0.5% uranyl acetate and 2% osmium tetroxide were used to stain the cells, followed by sequential dehydration and Epon-propylene oxide (Epon-PO) embedment. An ultramicrotome was employed to obtain ultrathin sections for Bio-TEM observation.

### Characterization of the nanoparticle uptake routes

The ZnCM NP uptake route was characterized using different route inhibitors (amantadine-HCl, 1 mM, MW 187.71; genistein: 200 μM, MW 270.24; amiloride, 1 mM, MW 302.12; cytochalasin, 2 μg/mL, MW 507.62). Nontoxic concentrations of ZnCM NPs were added to igDCs for 2 h and then examined with BD LSRFortessa equipment.

### Quantitative real-time PCR

TRIzol reagent was used to extract total RNA from cells. Nanodrop technology was used to detect the RNA concentration. One microgram of RNA was used for reverse transcription using a reverse transcription kit and a SYBR Green RT–qPCR master mix kit for real-time fluorescent quantitative PCR. The reaction conditions were as follows. Program 1: 95 °C, 30 s, 1 cycle. Program 2: 95 °C, 5 s and 60 °C, 34 s; 50 cycles. Program 3: 95 °C, 5 s, 1 cycle; 65 °C, 60 s; 97 °C, 1 s. Program 4: 42 °C, 30 s, 1 cycle. The relative expression of each gene was calculated according to the 2^−△△CT^ method, and the mRNA expression was normalized to the expression of GAPDH. The primers used are listed in Additional file 1: Table S1.

### Determination of the intracellular Zn^2+^ concentration and O_2_ homeostasis after nanoparticle treatment

For intracellular Zn^2+^ imaging, ZnCM NP-treated igDCs were washed and stained with 5 M FluoZin-3 AM for 20 min at room temperature. For excitation, light was filtered through a 470–495-nm excitation filter, and the emitted light was collected through a 525-nm filter. Images were acquired with a Leica SP8 confocal microscope. For intracellular O_2_ level determination, igDCs (1 × 10^6^/well) were seeded in glass bottom dishes (801,001, nest) for 12 h. Hypoxic cells and medium were induced as described previously [71]. Then, the cells were treated with the indicated dosage of ZnCM NPs for 6 h. The O_2_ indicator [Ru(dpp)_3_]_2_ + Cl_2_ (2 μM) was added. Intracellular O_2_ fluorescence was quantified by confocal microscopy. A reduction in red fluorescence intensity indicated a higher intracellular O_2_ level because oxygen quenches the fluorescence of this O_2_ indicator.

### Determination of dendritic cell activation after nanoparticle treatment

Flow cytometry and surface molecule marker staining were used to assess the activation of igDCs and T cells after ZnC and ZnCM NP treatments. The phenotypes of the igDCs and CFSE-labelled T cells were determined with a proliferation assay using flow cytometry as previously described [72]. First, igDCs were blocked with anti-CD16/32 on ice to exclude false-positive signals. Then, the cells were incubated with the appropriate antibodies for flow cytometry and CLSM analyses. For cytokine assays, the concentrations of IL-6, IL-12, p40, TNF, IL-17, and IFN-γ in igDCs were measured by CBA according to the manufacturer’s protocol (BD Biosciences). For determination of T-cell proliferation, igDCs were pulsed for 2 h with OVA peptide (200 nM) and then cocultured with OT-II CD4^+^ T cells (labelled with CFSE) and a 1:10 ratio of DC/T cells [49, 52]. After 3 days, T-cell proliferation was measured by flow cytometry to quantify the ratio of CFSE-low cells.

### Label-free quantitative proteomics

DC cells were divided into three groups: the Ctrl group (imDCs), vehicle group (LPS-induced igDCs) and ZnCM group (ZnCM-treated igDCs). The expression of proteins was verified by Wayen Biotechnology (Shanghai, China) according to their established protocols [73] using label-free quantification on an RPLC–MS/MS system. The data presented the most abundant proteins in the vehicle and ZnCM groups compared with the Ctrl group.

### Coimmunoprecipitation and western blotting

Cells were collected by centrifugation at 3000 rpm for 5 min, washed with PBS, added to an appropriate amount of modified RIPA buffer (containing protease inhibitor), and lysed on ice for 30 min. The supernatant was centrifuged at 4 °C for 20 min at 12,000 rpm, and a small amount of the supernatant was taken for western blot analysis as previously reported [74, 75]. The remaining supernatant was added to 1 μg of the corresponding antibody and incubated overnight at 4 °C. Prewashing of protein A beads: Ten microlitres of protein A beads was washed three times with an appropriate amount of modified RIPA buffer and centrifuged for 3 min at 3000 rpm each time, and then modified RIPA buffer was used to adjust the suspension to a volume ratio of 50%. Ten microlitres of prewashed protein A beads was then added to the modified RIPA buffer, incubated with neutralizing antibody overnight, and slowly shaken at 4 °C to fully couple the antibody with the protein A beads. After centrifugation at 4 °C and 3000 rpm for 3 min, the supernatant was discarded, and the beads were washed with 1 ml of modified RIPA buffer. Next, 15 μl of 2 × SDS sample loading buffer was added followed by boiling for 5 min. Western blotting analysis was used to determine the binding proteins.

### Collagen-induced rheumatoid arthritis mice, treatment, and X-ray/IVIS imaging

Adult female BALB/c mice (6 weeks old) were purchased from Shanghai SIPPR-Bk Lab Animal Co., Ltd. and kept in the specific-pathogen-free laboratory animal facilities of Shanghai Ninth People’s Hospital. All experimental procedures and animal care protocols were reviewed and approved by the Institutional Animal Care and Use Committee (IACUC) of Shanghai Ninth People’s Hospital. Collagen-induced arthritis (CIA) was induced by immunization with an emulsion of complete Freund’s adjuvant (CFA) and type II collagen (CII) as described previously [76]. Briefly, 50 μL of an emulsion containing CII and CFA at a ratio of 1:1 was injected intradermally into the end of the mouse tail. A booster immunization (CII and IFA, 1:1) was used 21 days after the primary immunization to ensure the induction of a high incidence of CIA. The onset of disease was confirmed on the day that swelling or erythema was observed in the hind paw, typically 4 to 7 days after the second immunization. Each experimental group (n = 6) was treated 4 times, 7 days apart, starting from Day 28 after the first immunization, with retrobulbar injection of saline (vehicle), ZnC NPs, or ZnCM NPs (100 μL per mouse) (indicated as 0 weeks). A healthy nonimmunized group was included as a control. Paw swelling was measured and recorded weekly. An IVIS (Lumina Series III, PerkinElmer) was used to measure the intensity of the enriched NPs in the organs in vivo.

### Bio-TEM in vivo

Small pieces of spleen specimens were fixed in glutaraldehyde for 1.5 h and then postfixed in osmium tetroxide (1%). Ultrathin Sects. (70 nm) were placed on copper grids, infused with uranyl acetate and lead citrate, and scoped. Next, Bio-TEM (FEI Talos L120C, Thermo Scientific) was performed to observe the intracellular distribution of the NPs in vivo.

### Visual gait analysis

To assess motor function and coordination in RA mice, gait analysis was performed with a VisuGait system (XR-FP101, Shanghai Xinruan). Briefly, mice walked across an illuminated glass platform with dark plastic walls spaced 5 cm apart in a dark room while a video camera recorded from below (120 frames/seconds). Trials in which the animal paced unevenly or changed direction were excluded from subsequent analysis. VisuGait analysis software (Version 2.0) was used to classify and measure the footprints by technical staff who were blinded to the mouse treatment.

### In vivo nanoparticle distribution

To assess the NP distribution in vivo, Zn^2+^ concentrations were used. The main organs from CIA mice were collected and broken down with aqua regia. Zinc ion concentrations were recorded with an Agilent 5110 ICP–OES instrument.

### µCT

We collected the tarsals and ankle joints, which were free of soft tissue, and fixed the specimens with 4% paraformaldehyde. Then, the specimens were analysed using a µCT-scan 80. The following settings were used: a resolution of 9-µm voxel size, a voltage of 70 kV, a current of 200 µV, and 200 ms of integration time. Three-dimensional images were reconstructed and analysed by defining the whole ankle joint as the region of interest (ROI). The three-dimensional structural parameters BV/TV, Tb.Th, Tb.N and BMD were analysed.

### Histology, immunohistochemistry, and immunofluorescence

Spleen and ankle samples were sectioned to obtain 4-μm-thick slices. The slides were blocked with 5% normal goat serum in PBS containing 20 mM glycine for 1 h followed by incubation with the indicated antibodies overnight at 4 °C. Sections were then washed and incubated with the appropriate secondary antibody for 45 min to acquire histological and immunohistochemical images. For immunofluorescence, slides were mounted with Dako Fluorescence Mounting Medium (#S3023, Agilent Dako). A Leica SP8 confocal microscope was used for confocal imaging of the samples. Leica Application Suite X software was used for analysis.

### Statistical analysis

SPSS (Statistical Package for the Social Science) 13.0 software was used to analyse the collected data, which are presented as the means ± standard deviations. Homogeneity was confirmed with a comparison of variance test. Parametric data were analysed using one-way analysis of variance followed by a post hoc Tukey’s test to compare the two groups. A P value ≤ 0.05 indicated a significant difference.

## Supplementary Information

**Additional file 1: Figure S1.** (a) XRD pattern of ZnO2 NPs. (b) Zn2+ released from ZnO2 (100 μg/mL) NPs under different pH values. (c) H2O2 released from ZnO2 (1 mg/mL) NPs under different pH values. **Figure S2.** Colocalization of ZnCM NPs within DCs lysosomes. **Figure S3.** Quantitative intracellular hypoxia state in DCs before and after various NPs treatments. ** indicated the significant difference of p≤0.05. **Figure S4.** Repression of igDCs (signal 1,2) after NPs treatment. CLSM observations of igDCs molecules after various NPs treatments. **Figure S5.** Repression of igDCs (signal 3) after NPs treatment. **Figure S6.** Repression of T cells by tDCs after NPs treatment. **Figure S7.** Quantification of expressed proteins in DCs after NPs treatments. **Table S1.** Primers lists.

## Data Availability

The data and materials are available from the corresponding authors upon reasonable request.
