# Supplementary material for: Immune-regulating strategy against rheumatoid arthritis by inducing tolerogenic dendritic cells with modified zinc peroxide nanoparticles
Source: J Nanobiotechnology. 2022 Jul 14;20:323. doi: 10.1186/s12951-022-01536-0 (PMC9281050; doi:10.1186/s12951-022-01536-0)
Supplement: Supplementary file 1 — Additional file 1: Figure S1. (a) XRD pattern of ZnO2 NPs. (b) Zn2+ released from ZnO2 (100 μg/mL) NPs under different pH values. (c) H2O2 released from ZnO2 (1 mg/mL) NPs under different pH values. Figure S2. Colocalization of ZnCM NPs within DCs lysosomes. Figure S3. Quantitative intracellular hypoxia state in DCs before and after various NPs treatments. ** indicated the significant difference of p≤0.05. Figure S4. Repression of igDCs (signal 1,2) after NPs treatment. CLSM observations of igDCs molecules after various NPs treatments. Figure S5. Repression of igDCs (signal 3) after NPs treatment. Figure S6. Repression of T cells by tDCs after NPs treatment. Figure S7. Quantification of expressed proteins in DCs after NPs treatments. Table S1. Primers lists. [file 12951_2022_1536_MOESM1_ESM.pdf]

Immune-regulating strategy against rheumatoid arthritis by inducing tolerogenic dendritic cells with modified zinc peroxide nanoparticles

Han Qiao[1,2,#], Jingtian Mei[1,2,#], Kai Yuan[1,2], Kai Zhang[1,2], Feng Zhou[3], Tingting Tang[1,2,*], Jie Zhao[1,2,*]

1. Department of Orthopaedic Surgery, Shanghai Ninth People's Hospital, Shanghai Jiao Tong University School of Medicine, Shanghai, China PR.

2. Shanghai Key Laboratory of Orthopaedic Implants, Shanghai Ninth People's Hospital, Shanghai Jiao Tong University School of Medicine, Shanghai, China PR.

3. Department of Orthopaedic Surgery, The First Affiliated Hospital of Soochow University, Suzhou, Jiangsu Province, China PR.

# These authors contributed equally to this work.

*Corresponding to:

Jie Zhao, Ph.D., M.D., Department of Orthopaedics, Shanghai Ninth People's Hospital, Shanghai Jiao Tong University School of Medicine, Shanghai Key Laboratory of Orthopaedic Implants, 639 Zhizaoju Road, Shanghai 200011, China PR. E-mail, profzhaojie@126.com;

Also corresponding to:

Tingting Tang, Ph.D., M.D., Department of Orthopaedics, Shanghai Ninth People's Hospital, Shanghai Jiao Tong University School of Medicine, Shanghai Key Laboratory of Orthopaedic Implants, 639 Zhizaoju Road, Shanghai 200011, China PR. E-mail, ttt@sjtu.edu.cn

Supplementary Information

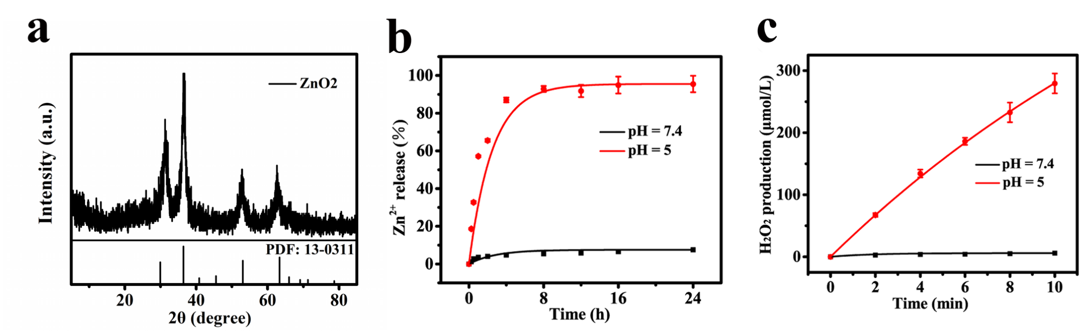

Supplementary Figure 1. (a) XRD pattern of ZnO_2 NPs. (b) Zn^{2+} released from ZnO_2 (100 μg/mL) NPs under different pH values. (c) H_2O_2 released from ZnO_2 (1 mg/mL) NPs under different pH values.

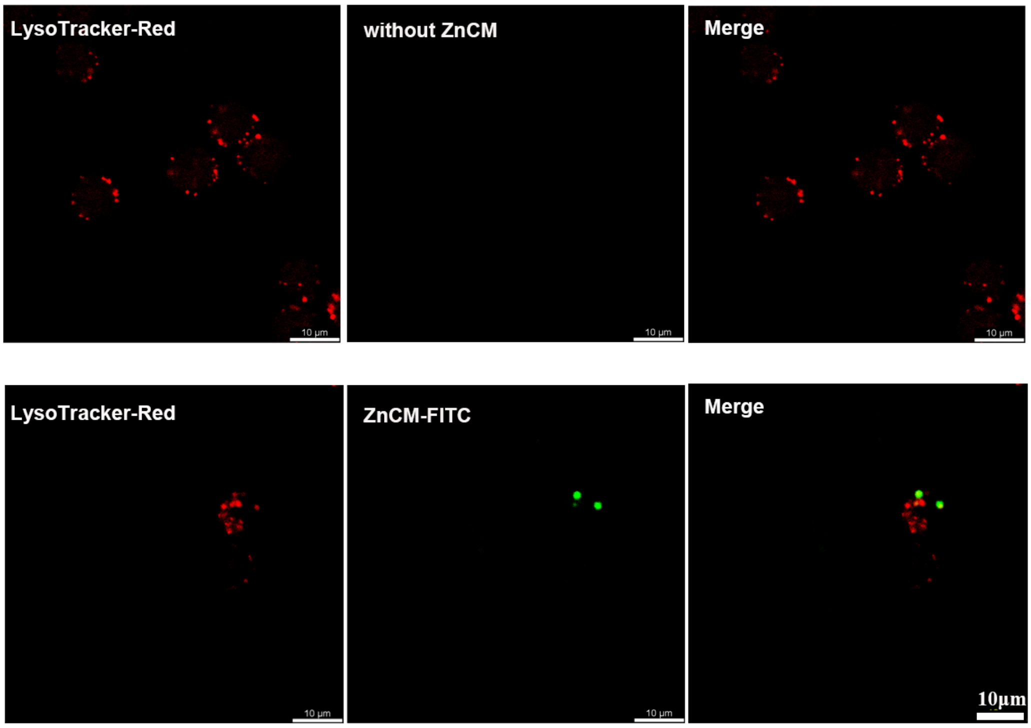

Supplementary Figure 2. Colocalization of ZnCM NPs within DCs lysosomes. CLSM observations after DCs were treated with non-toxic fluorescent ZnCM NPs.

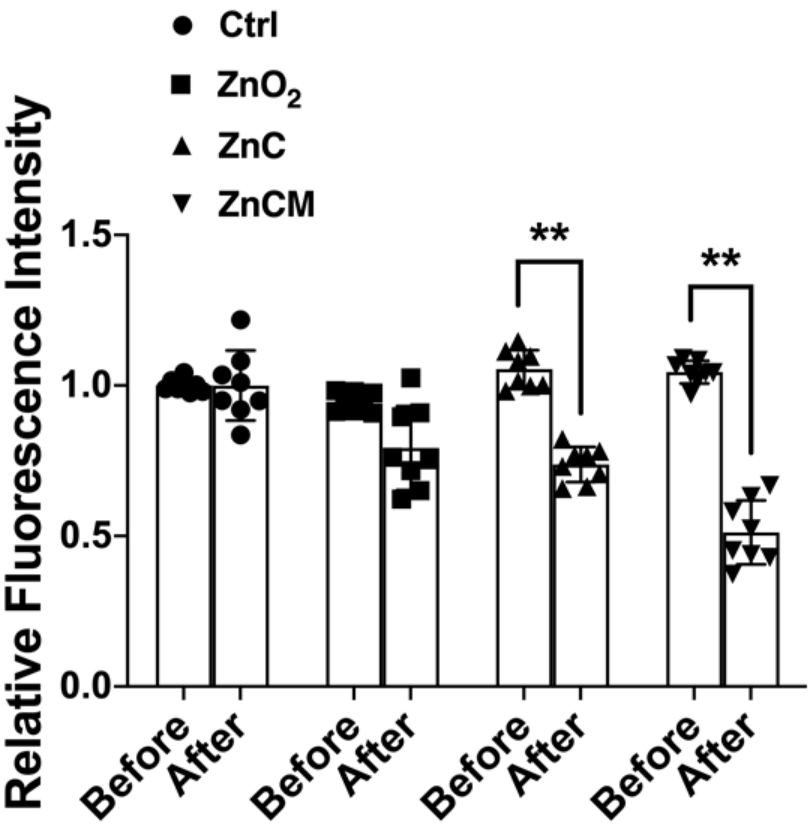

Supplementary Figure 3. Quantitative intracellular hypoxia state in DCs before and after various NPs treatments. ** indicated the significant difference of $p \le 0.05$.

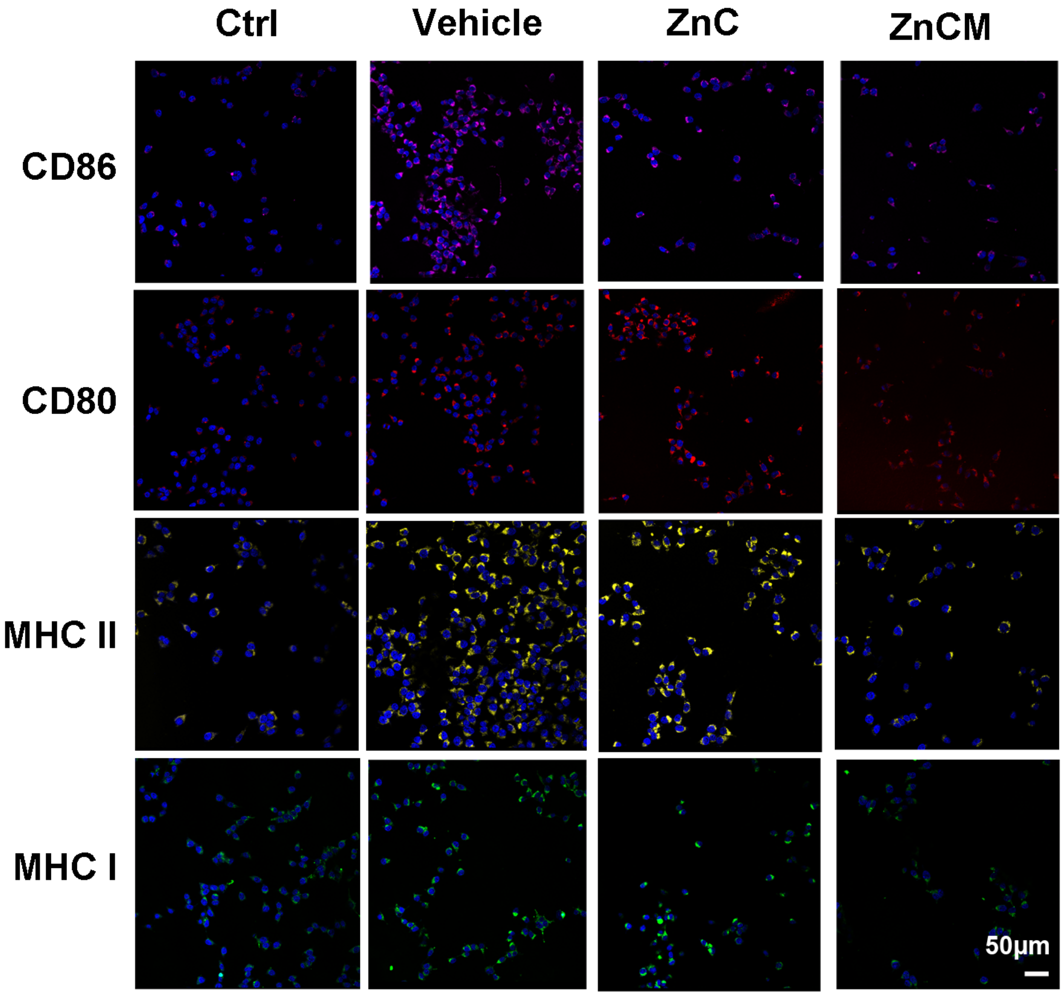

Supplementary Figure 4. Repression of igDCs (signal 1,2) after NPs treatment. CLSM observations of igDCs molecules after various NPs treatments.

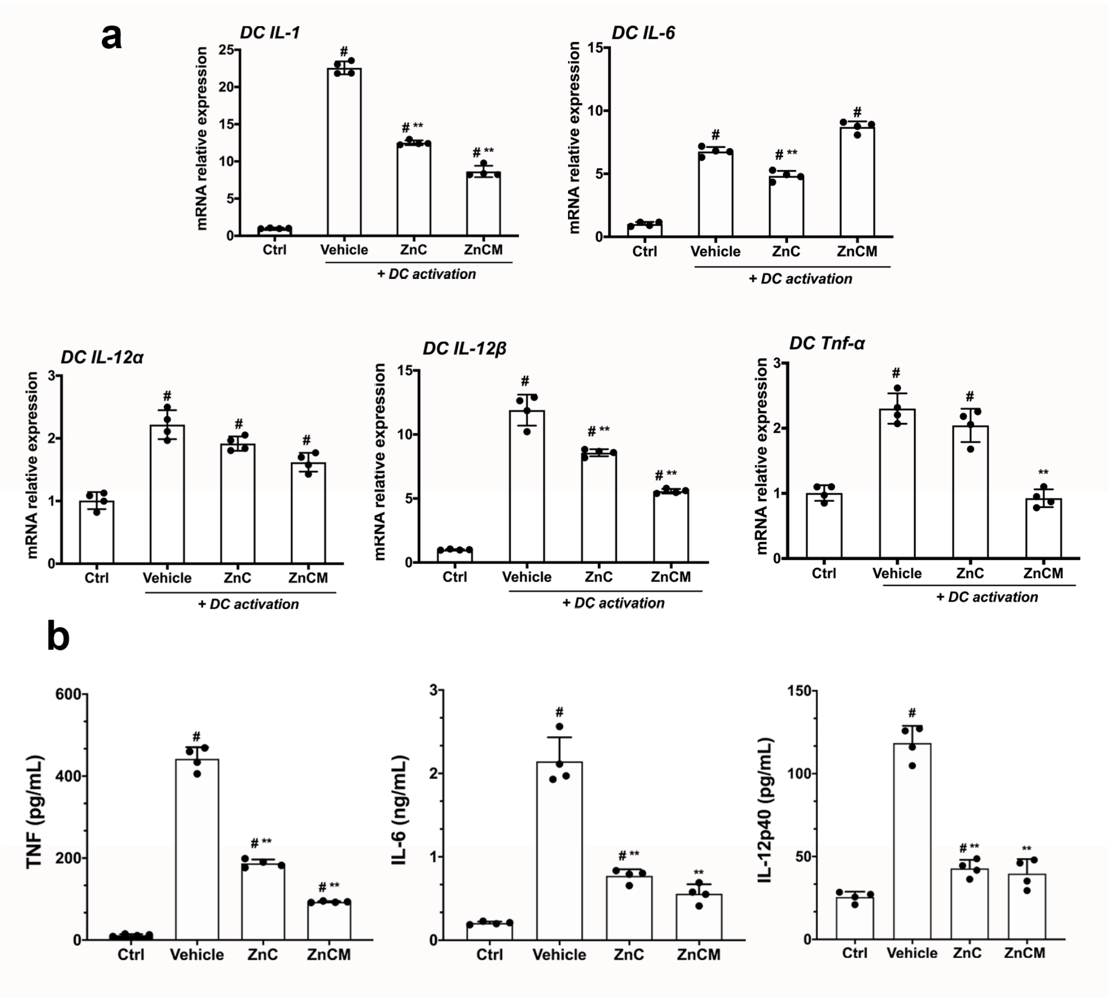

Supplementary Figure 5. Repression of igDCs (signal 3) after NPs treatment. (a) mRNA level of immune cytokines from igDCs treated with NPs towards T cell homeostasis regulation. (b) Cytometric bead array (CBA) immunoassay analyzing the levels of TNF, IL-6, and IL-12 from igDCs treated with NPs. ** indicated the significant difference of $p \le 0.05$ compared with Vehicle, # indicated the significant difference of $p \le 0.05$ compared with Ctrl.

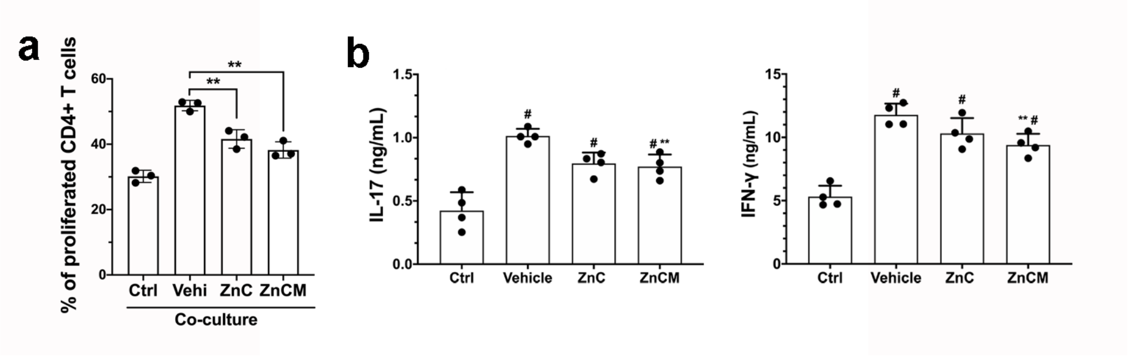

Supplementary Figure 6. Repression of T cells by tDCs after NPs treatment. (a) Proliferation of OT-II CD4$^+$ T cells cocultured with DCs treated with various NPs assessed by

flow cytometry. ** indicated the significant difference of p≤0.05. (b) CBA results showed the release of IL-17 and IFN-γ from CD4+ T cells after co-culture with igDCs treated with NPs. ** indicated the significant difference of p≤0.05 compared with Vehicle, # indicated the significant difference of p≤0.05 compared with Ctrl.

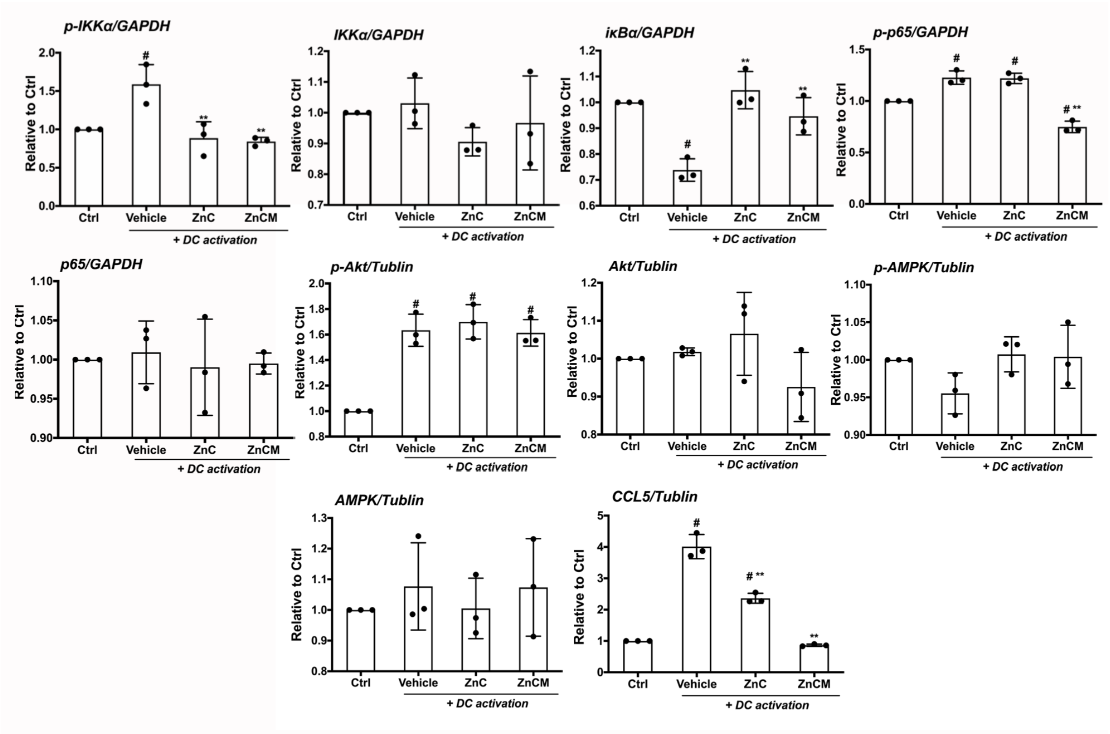

Supplementary Figure 7. Quantification of expressed proteins in DCs after NPs treatments. ** indicated the significant difference of p≤0.05 compared with Vehicle, # indicated the significant difference of p≤0.05 compared with Ctrl.

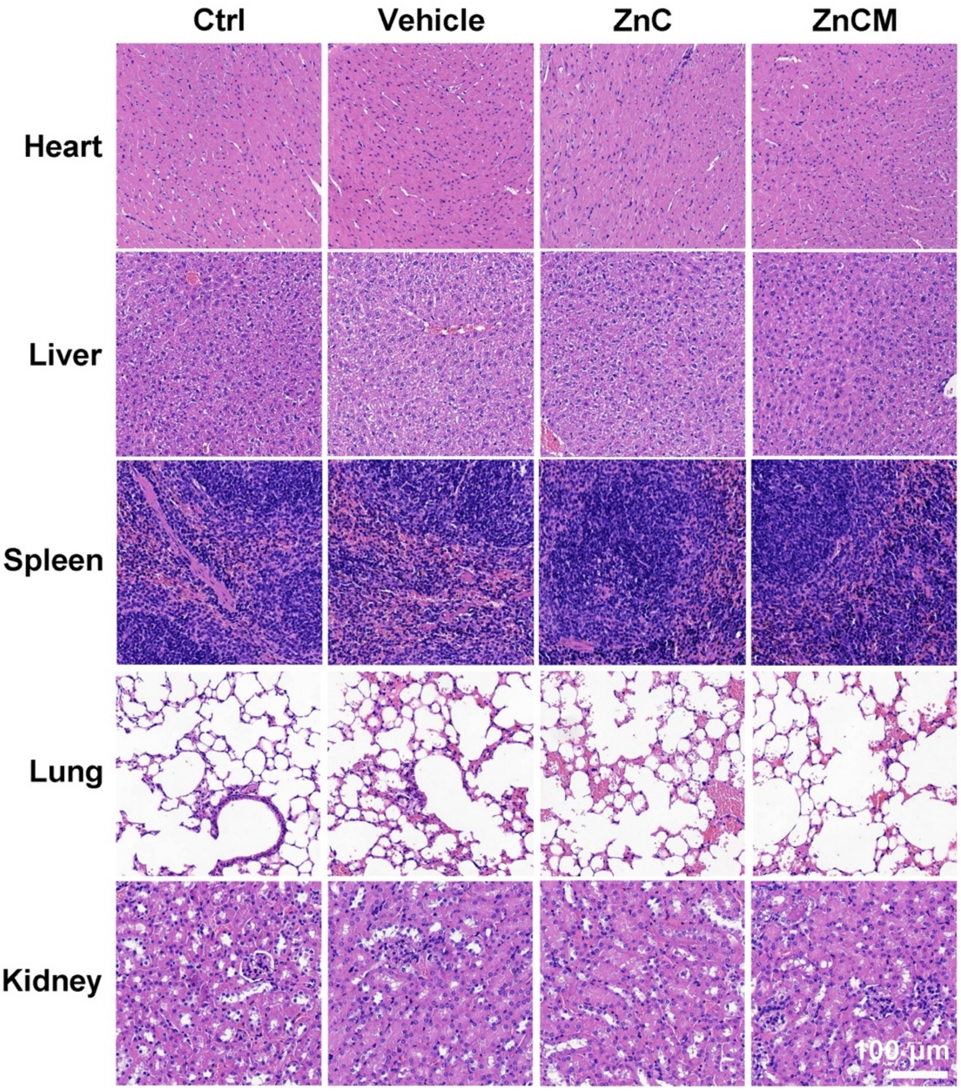

Supplementary Figure 8. Biosafety of NPs. The HE histologic images of heart, liver, spleen, lung, and kidney in RA mice injected with varying NPs for 4 weeks.

Supplementary Table 1. Primers lists.

Primer	5' to 3'
UCHL3F	GCCCTGAAGAGAGAGCCAAA
UCHL3R	GGTGCCTCAGTCTGACCTTC
UCHL5F	ACTCAGTGCACGTTTGGCAT
UCHL5R	TGAAGAATGGAGTTTAGCCAGC
BAP1F	CCAAACGCCAGTGAGAACCT
BAP1R	ACATGTCACTCCCACTCCCA
UCHL1F	AGTCTGGGGAGAGAGAACCA
UCHL1R	GGGGCTGTAGAACGCAAGAA
USP15F	TCCCTCTACTCCTAATGTGAAAAAC
USP15R	AGGCCTGGCTGTTCATTGTT
USP46F	CTTCCCCCATCACACAGCTC
USP46R	GCAGGTCTCAACTCCACCTG
USP14F	CGGGAAATTACGTTTGGGTCC
USP14R	CTTGGGCTGAAAAGGCAAGG
USP21F	GAAGCCGGTGGCCGGT
USP21R	CCCCTCTTATCCCCAAACAGC
USP49F	AGAACATCAGTGCCCACAGG
USP49R	GCCACAAGCAAACGATCCAG
USP38F	GGCGTTGCACTCCAGATTCA
USP38R	TTAAAACCCCTGCCCTCTGG
USP4F	AGCCTGCAGTCAAATGGATCT
USP4R	GGCCAGGCTGTATATGAGGTG
USP25F	TCAATGAAGTGATGTTGTGAATGA
USP25R	CAAAAATAGATCGGTGGTAACATGA
USP44F	AGCATGTGCACGATGGAAGA
USP44R	TATTGGACAGGAGCTCGGGA
USP22F	TTGGTCTTTTTGCCTGCCCA
USP22R	GTCTCCAACTGGACCTCAGC
USP3F	TCAGCCTTTACAGCGGACAG
USP3R	CTACTAAAGACCTGAGTGACTGAA
USP26F	TCTAGATCCGTGGGGGTCTG
USP26R	CGGTCCTCACTCTGGTTCAC
USP5F	GGGAGACTGGCTACCCCTTA
USP5R	CTGGGGTCCAGAACCATGTC
USP13F	ACATTGAGGAGTTACCAGCCC
USP13R	ACTGGCACTTCGTTTTCCCA
USP12F	AGAAAGGATCGAGCGGATGT
USP12R	CAAAGTGAGCTACTGCCCTGA
USP18F	AGGGCGGCTGTGCCTA
USP18R	AATGACCCTCTCAAGCACGG

**USP39F**	GCTCCCTTCATCTCCTGTGG
**USP39R**	ACCCTTAACAAGCCTCAATGGG
**USP27XF**	GTGTTCTGGATGGGGAAGGG
**USP27XR**	TATCCTCGCCTCCTCCATGT
**USP8F**	ATATACACAGCGCGCAGAAG
**USP8R**	GCCCTTTCCTCATCACGATCT
**USP33F**	CCACACCTGCCAAATTGAGTT
**USP33R**	TGGACCTGGGGGATCCTTTA
**PAN2F**	CAGAGGTGGAACCGCTTCAT
**PAN2R**	TGAGCAGAAACTGCCTCCAG
**USP20F**	GCACTACTGTGACGCAGTCT
**USP20R**	AGTACAAGCTAGGGGCAGGA
**USP45F**	GAAGCTCCTGCGTGTGGAAG
**USP45R**	ATCATGAGGCAGCAGTGGTC
**USP48F**	ATCCCAGACCCGTAAACCCA
**USP48R**	CCTGGGCTTGCAGCCTATAA
**USPL1F**	ACAGCTCTCCACCATTGAGC
**USPL1R**	CCTTCCTGCTCCCTCAACAG
**USP1F**	GAAGGGCCATGACCGGATTT
**USP1R**	GACTGCCTCTTGAAAGCCCA
**USP2F**	CTCCATCCAGTGCCCCCAAC
**USP2R**	CCATAGCCCGGTTTGGCAT
**USP10F**	TTTGTGACTCCCCGCTCTTC
**USP10R**	CTGGTGCTCCTGTCCATCTG
**USP36F**	CGAAGGCTGACAGTCCAGG
**USP36R**	TCTCTGCAGCAGGTAAGGGA
**SENP2F**	CTGCTCAGGCCTGGAATGT
**SENP2R**	CAGCTGGAACGGGAATCCAA
**CYLDF**	CAGGTAGCAGGTTCGGCTG
**CYLDR**	ACTGGCAAAAAGGAGCCACT
**YOD1F**	TCAGACCTGTCCTCTGGGTT
**YOD1R**	TGCTCAAGTGGTAAAGGTAAGC
**OTUD3F**	GCTTAATGCCCCTTTGTGGC
**OTUD3R**	CCGGACGCTGTCATAGTGTT
**OTUB2F**	TCTGCCACCTAGGTCCTTCC
**OTUB2R**	GTGTCCTTGCCTCAGGTCTC
**OTUD6AF**	TCGAGAATATGCCTCCACGC
**OTUD6AR**	GCGACCTTTTCCTCCTCCTC
**TNFAIP3F**	CCTGCCAGCAGGTATATGGG
**TNFAIP3R**	CAAGGCCTGAAGAGGAAGGG
**OTUB1F**	TCCCCAGCTTTCCAACCATC
**OTUB1R**	GCCTATGGGAGCAGAACTCC

**ZRANB1F**	CCGCGGGGTTTATTTAGCTC
**ZRANB1R**	AGTCAGGACATGTTCTCCGC
**ALG13F**	CCTTTAATTCTAACCTTTGGGAGCC
**ALG13R**	CTCTGGGTAGAAAAGGATGGC
**ATXN3F**	CCCTCAAAGCAGACCTGGAG
**ATXN3R**	TACTGAGCTGAATGGCCCTG
**JOSD1F**	CACTGGCCTGCTTCTTCTCC
**JOSD1R**	CGAGGCCTCTGCCACTTG
**JOSD2F**	CCATTGCTAGCAGGAGCTGA
**JOSD2R**	CTTGCAGATTTCGTCGGCAG
**STAMBP1F**	TTCACGAAAGGTGGGAAGGG
**STAMBP1R**	AAGGAGTGTTGAGACCAGGC
**PSMD14F**	AAAGGAAGCCGAAGGAAGCC
**PSMD14R**	CTCGGAGAACAGGCGATGAA
**BRCC3F**	GGAGCAAAACCAGCAGCATT
**BRCC3R**	GCCAGGTATTTTCATCTGCCC
**COPS5F**	GCCTTGAGAGTCTATCACCACT
**COPS5R**	TGATGATCATGGTCTCGCCG
**STAMBPL1F**	CATCCTCACACCAAGGACCC
**STAMBPL1R**	TCACACACGTCCACAGATGG
**EIF3FF**	TCGAGGTTTTCACCACAGGG
**EIF3FR**	ATGCTTCTTTCTCCTGGCCG
**UFSP1F**	TGGGCCTGGCACAATGATAC
**UFSP1R**	CAGCTTGGCCTTGCTGTGTA
**SENP5F**	CCGGGAGCAAAGAACGTACA
**SENP5R**	GCTTCCAAACCCAGCATTCC
**SENP8F**	ACAAAGAATCCGCAATGCCC
**SENP8R**	ACTCAAGACCACAGGGTCCA
**SENP7F**	CGCGAATCTCACTTCTGGCT
**SENP7R**	ATCTCTGATGAAGCTCGCCG
**SENP1F**	GAGTAAAGAAGGTTCCGGTTCCC
**SENP1R**	CCGCCACTCACCGAACC
**SENP3F**	TGGCCTAGACGCGCTGA
**SENP3R**	CCAAAACCACCACCGGACT
**ZC3H12AF**	TGCTGTGTACAGAGGCGAGA
**ZC3H12AR**	CCACCATGTGGTTGCTGACA
**UFD1LF**	CAGTGCAGCATGAGGAGTCA
**UFD1LR**	GAACCAGAGAAGGCACGGAA
**TAF1DF**	ACTTGCTGGTGGAACGCTAA
**TAF1DR**	CTGCAGATGAGCAAAAGGGC
**TRIM44F**	ATGCCCATTCACACTCGGAA
**TRIM44R**	TCCTGGAAGGAACAATGAATACT

**IL-1β**	TTCAAGGGGACATTAGGCAG
**IL-6**	CAACGATGATGCACTTGCAGA
**IL-12α**	CTGTGCCTTGGTAGCATCTATG
**IL-12β**	GTGGAATGGCGTCTCTGTCT
**TNF-α**	CTCAGCGAGGACAGCAAGG
